# Adenylyl cyclase isoforms 5 and 6 in the cardiovascular system: complex regulation and divergent roles

**DOI:** 10.3389/fphar.2024.1370506

**Published:** 2024-04-03

**Authors:** Saeid Maghsoudi, Rabia Shuaib, Ben Van Bastelaere, Shyamala Dakshinamurti

**Affiliations:** ^1^ Department of Physiology and Pathophysiology, University of Manitoba, Winnipeg, MB, Canada; ^2^ Biology of Breathing Group, Children’s Hospital Research Institute of Manitoba, Winnipeg, MB, Canada; ^3^ Section of Neonatology, Department of Pediatrics, Health Sciences Centre, Winnipeg, MB, Canada

**Keywords:** adenylyl cyclase, G protein-coupled receptors, cyclic 3′,5′-adenosine monophosphate, signal transduction, heart disease, drug discovery

## Abstract

Adenylyl cyclases (ACs) are crucial effector enzymes that transduce divergent signals from upstream receptor pathways and are responsible for catalyzing the conversion of ATP to cAMP. The ten AC isoforms are categorized into four main groups; the class III or calcium-inhibited family of ACs comprises AC5 and AC6. These enzymes are very closely related in structure and have a paucity of selective activators or inhibitors, making it difficult to distinguish them experimentally. AC5 and AC6 are highly expressed in the heart and vasculature, as well as the spinal cord and brain; AC6 is also abundant in the lungs, kidney, and liver. However, while AC5 and AC6 have similar expression patterns with some redundant functions, they have distinct physiological roles due to differing regulation and cAMP signaling compartmentation. AC5 is critical in cardiac and vascular function; AC6 is a key effector of vasodilatory pathways in vascular myocytes and is enriched in fetal/neonatal tissues. Expression of both AC5 and AC6 decreases in heart failure; however, AC5 disruption is cardio-protective, while overexpression of AC6 rescues cardiac function in cardiac injury. This is a comprehensive review of the complex regulation of AC5 and AC6 in the cardiovascular system, highlighting overexpression and knockout studies as well as transgenic models illuminating each enzyme and focusing on post-translational modifications that regulate their cellular localization and biological functions. We also describe pharmacological challenges in the design of isoform-selective activators or inhibitors for AC5 and AC6, which may be relevant to developing new therapeutic approaches for several cardiovascular diseases.

## Introduction

Over the course of life, heart health and aging have been correlated with diminished left ventricular (LV) function and weakened cardiac β-adrenergic receptor (βAR) responsiveness. The reasons for this dysfunctionality may include phenotype changes in the LV, reduced βAR density, upregulation of regulatory proteins Gαi and G-protein coupled receptor kinases, or abnormalities in the βAR signaling system (Ferrara et al., 2014). Adenylyl cyclases (ACs), as the central effector molecules for βAR signaling, play a crucial role in cardiac contractility, relaxation, and LV diastolic function (Tang et al., 2011). The failing heart has poor AC signaling and decreased LV cAMP production, leading to impaired βAR responsiveness to ligands (White et al., 1994; Roth et al., 2002). Persistent activation of the sympatho-adrenergic system in patients with congestive heart failure can also lead to unfavorable cardiac remodeling due to cardiomyocyte loss and fibrotic replacement (Zhang et al., 2013). The distribution of AC isoforms varies within cardiac tissues; ACs 2, 3, 4, 5, 6, and 7 are expressed by cardiac fibroblasts (Ostrom et al., 2003), while AC1 and AC8 are found in sinoatrial node (SAN) cells (Mattick et al., 2007; Younes et al., 2008; Robinson et al., 2021), and AC5 and AC6 are the major AC isoforms expressed in the adult ventricle (Göttle et al., 2009; Efendiev and Dessauer, 2011). AC6 also serves as the principal effector of vasodilator signaling and a regulator of membrane potential in vascular myocytes (Nelson et al., 2011). In this review, we examine the similarities and differences between ACs 5 and 6 in cardiovascular function, highlighting their divergent functional roles and opportunities for pharmacological targeting.

## Overview of adenylyl cyclases

**FIGURE 1:**
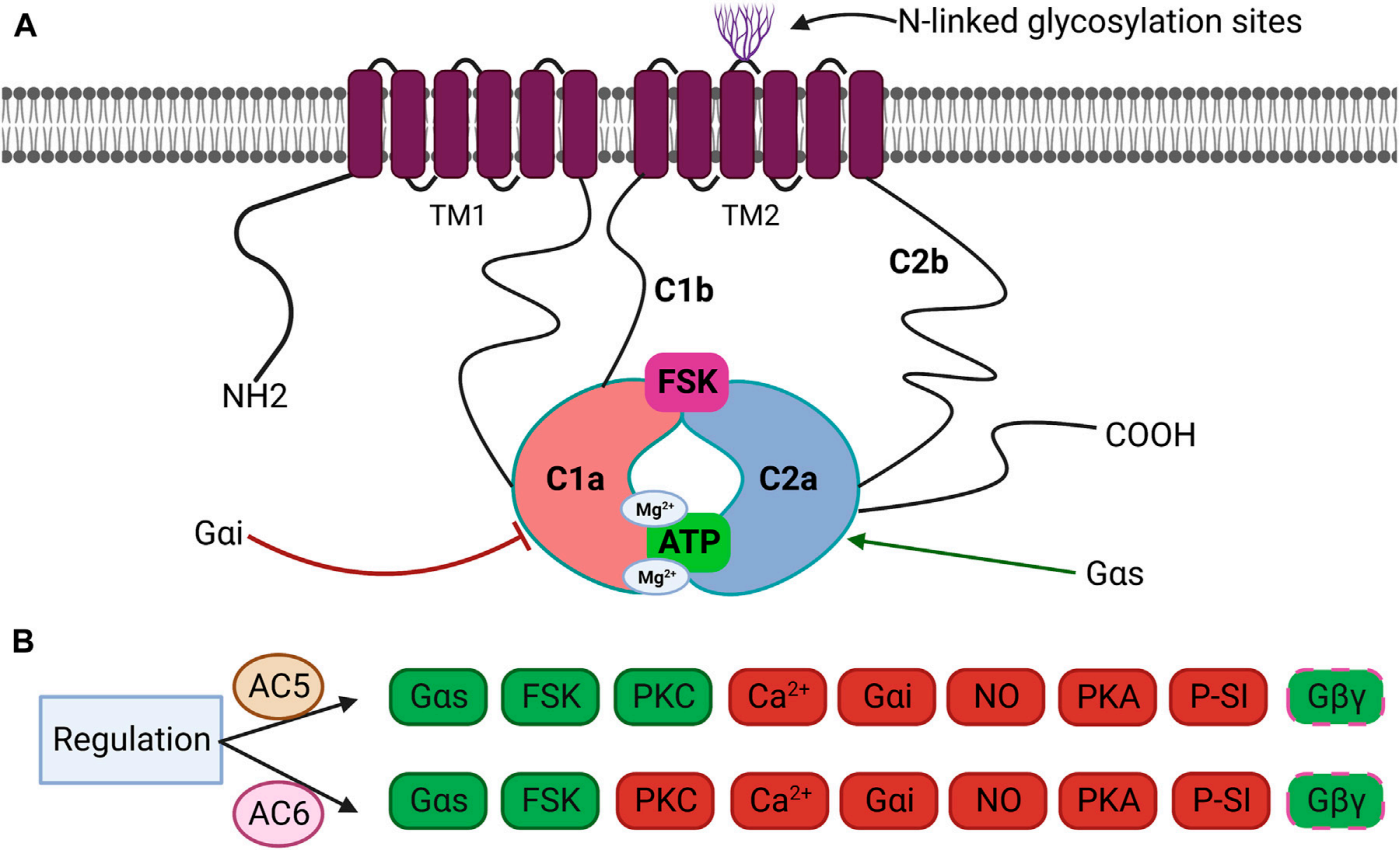
**(A)** Schematic structure of transmembrane adenylyl cyclases (TMACs). TMACs contain an intracellular N-terminus, two repetitions of six TM1 and TM2, and two cytoplasmic domains (C1 and C2) further divided into C1a and C2a. C1a–C2a forms the catalytic domain and FSK-binding site. Inhibitor Gαi binds to C1a, whereas activator Gαs binds to C2a. C1b and C2b are regulatory subdomains, and the N-terminus participates in several protein–protein interactions. Some isoforms are glycosylated on extracellular loops 5 or 6. **(B)** Specific regulation of AC5 and AC6 by various effectors. Both isoforms are fully activated by Gαs and FSK and partially or conditionally activated by Gβγ. AC5 is activated by PKC, whereas AC6 in inhibited by PKC. Both isoforms are inhibited by Ca^2+^, Gαi, NO, PKA, and P-site inhibitors (P-SI). Green: stimulation, red: inhibition, and dashed line: conditionally.

Cyclic 3′,5′-adenosine monophosphate (cAMP), a ubiquitous second messenger that mediates a variety of cellular responses, is generated by more than thousand nucleotidyl cyclase proteins classified into six groups according to the amino acid sequence of the catalytic domain (Kamenetsky et al., 2006; Seifert and Beste, 2012). The class III cyclases including eukaryotic adenylyl cyclases (ACs) are indispensable effectors of cAMP (Linder, 2006). Enumerated in order of discovery, mammalian ACs 1–9 (_∼_120–140 KDa) have been identified as transmembrane adenylyl cyclases (TMACs), while AC10 is a soluble enzyme. ACs are canonically regulated by heterotrimeric G-proteins, Gαi and Gαs, upon stimulation of G protein-coupled receptors (GPCRs) (Halls and Cooper, 2017). There are significant variations in the distribution and biochemical characteristics among AC isoforms, as well as distinctive chromosomal loci of individual isoforms (Vatner et al., 2013). The TMACs share a similar framework but different regulation (Sunahara et al., 1996). Despite arising from distinct transcripts, they own a similar overall structure with highly homologous active sites, making it challenging to design selective activators or inhibitors and to differentiate their functions in the organs and tissues (Rana et al., 2017). The mammalian ACs share an intracellular N-terminus, two membrane-spanning domains (TM_1_ and TM_2_), and conserved catalytic domains, C1 and C2 (each _∼_40 KDa), together forming the catalytic core. The C1 and C2 cytoplasmic domains are further divided into the highly conserved catalytic regions, C1a and C2a, and the less conserved regulatory regions, C1b and C2b. Two active sites are shaped by their interface: the ATP catalytic site and forskolin (FSK) binding pocket (Figure 1A) (Schmid et al., 2014; Antoni, 2020). Crystallographic studies have revealed that the hydrophobic pocket generated by C1 and C2 catalytic subunits is the allosteric site for the interaction of FSK with ACs (Pavan et al., 2009; Bhatia et al., 2023). Despite the sequence similarity of the regulatory domains in C1–C2, various signals such as G-proteins, kinases, FSK, and Ca^2+^ uniquely regulate AC isoforms (Brand et al., 2013). Indeed, the C1–C2 domain, at the cytoplasmic surface of the molecule, accommodates binding sites for Gαs, Gαi, FSK, ATP, Mg^2+^, the regulator of G protein signaling (RGAS2), proteins associated with Myc (PAM), Snapin, Ric8a, A-kinase-anchoring protein (AKAP79), PH domain leucine-rich protein phosphatase 2 (PHLPP2), and phosphorylation and dephosphorylation sites for protein kinase A (PKA) and protein kinase C (PKC) (Sunahara et al., 1997). The crystal structures of the complex made up of the C1 cyclase homology domain (CHD) from AC5 and the C2 CHD from AC2 (VC_1_:IIC_2_) bound to FSK and GTP-S-activated Gαs served as the fundamental model for the prototype AC catalytic core (Mou et al., 2009). Binding sites for cofactors Mg^2+^ and Mn^2+^ are further located in the active site of AC and must be occupied for catalytic activity, based on structural analyses of activated VC_1_:IIC_2_ complexes coupled to substrate analogs (Sinha and Sprang, 2006; Mou et al., 2009). Forskolin, a natural diterpene, specifically binds opposite to the ATP-binding site within the catalytic core, using a mixture of hydrophobic and hydrogen interactions to approximate the two cytoplasmic domains, resulting in increased enzyme activity regardless of Gαs docking. No endogenous ligand has yet been identified for the FSK-binding site (Pavan et al., 2009; Dessauer et al., 2017; Bhatia et al., 2023).

While the C1–C2 framing the catalytic core remains highly conserved among individual AC isoforms, the N-terminus is significantly varied across isoforms (Antoni, 2020). The length and composition of the N- and C-terminal domains of ACs are significantly variable outside of the non-conserved domains (Sunahara et al., 1996). The N-terminus of AC1 to AC5 spans 60 to 240 residues in length. Pharmacologically, these are the regions where type-specific regulation of ACs can occur (Halls and Cooper, 2017). The TM domains are not required for catalytic activity since C1a and C2a can be individually shaped into active enzymes by Gαs and FSK (Tang and Gilman, 1995). Removing TM domains and fusing just C1 with C2 make a small but versatile protein which can be used to replicate numerous beneficial effects of AC (Tan et al., 2019). However, the TM regions play important roles in balancing the stoichiometry of their relative catalytic domains (Sunahara et al., 1996), in regulating functional assembly and trafficking of AC (Gu et al., 2002), and may also function as receptors for extracellular signals (Seth et al., 2020).

From a regulatory standpoint, the mammalian AC isoforms are classified into four groups: *Group I*, ACs 1, 3, and 8, stimulated by calcium/calmodulin (Ca^2+^/CaM); *Group II*, ACs 2, 4, and 7, stimulated by Gβγ; *Group III*, ACs 5 and 6, inhibited by Gαi/Ca^2+^; and *Group IV*, AC9, which is partially stimulated by FSK (Ostrom et al., 2022). All isoforms are activated by Gαs while differentially regulated by Gαi, Gβγ, and protein kinases (PKA, PKC, Raf-1, and CaM kinases) (Figure 1B) (Antoni, 2020). Most cells and tissues express multiple AC isoforms; however, their varying abundance in specific tissues is noteworthy (Halls and Cooper, 2017; Ostrom et al., 2022).

## Role of ACs in cardiac tissue

Key cardiac isoforms AC5 and AC6, known as the Ca^2+^-inhibited family of ACs, share about 91.5% sequence alignment, except for the AC6 N-terminus (aa 1–86) (Wu et al., 2017). These two isoforms have considerable similarities in their expression patterns and functions, despite their evolution at independent gene loci; the AC5 gene is on chromosome 3 at position 3q13.2–3q21, and AC6 is on chromosome 12 at position 12q12–12q13 (Haber et al., 1994). The canine (type V-a) and the rat (type V-b) forms of AC5 are in fact mRNA splice variants, co-expressed in both species (Iwami et al., 1995). Both AC5 and AC6 are non-competitively inhibited by Gαi at C1a, opposite from the docking site for Gαs at C2a (Taussig et al., 1994). Their tissue distribution and developmental mRNA expression patterns have been studied in several species, including humans (Tobise et al., 1994; Espinasse et al., 1995; Wang and Brown, 2004). AC6 is more expressed in the neonatal heart, while AC5 appears to be predominantly an adult isoform (Espinasse et al., 1995). The maximal amount of AC6 mRNA is expressed during fetal development, while steadily diminishing with age, and reaching its lowest amount in fully mature adults. On the contrary, a minimal amount of AC5 mRNA is expressed during fetal development, but progressively increasing with age, and reaching its highest point in the fully developed adult (Tobise et al., 1994; Okumura et al., 2009). In the mouse heart, the abundance of AC5 protein reaches its highest point before 1 week and then decreases to levels similar to those of mice studied at 3, 6, and 24 months of age. In the rat heart, however, the AC5 protein level peaks before 2 weeks of age and subsequently decreases by 3 months. In pigs, cardiac AC5 protein abundance is high in 1-day-old hearts and then diminishes with age, reaching its lowest content in the heart at 5–6 months (Hu et al., 2009). Feedback regulation of AC expression also varies as a function of age; PDE3 inhibition (PDE3i) has been found to alter AC expression levels in adult and pediatric subjects with dilated cardiomyopathy (DCM) and in pediatric subjects with a congenital single right ventricle. These groups demonstrated distinctive AC isoform mRNA expression patterns. Compared to the non-failing adult LV myocardium, the adult DCM myocardium exhibits upregulation of AC6; chronic PDE3i treatment enhances mRNA expression of AC5 and AC6, while all other isoforms are expressed at levels comparable to those of DCM patients not receiving PDE3i treatment. In contrast, the non-failing pediatric right ventricle expresses less AC5 than does the pediatric single ventricle; and in pediatric DCM, AC5 and AC6 remained unchanged by PDE3i (Nakano et al., 2017). Due to their structural homology and the paucity of type-specific antibodies, definitive levels of protein expression in the heart have been difficult to establish (Hu et al., 2009). Even following the deletion of AC5, 60% of immunodetectable AC5/6 was observed in cardiac myocytes, suggesting some degree of epitope overlap (Okumura et al., 2003b).

To distinguish their specific roles in cardiac function, AC5 and AC6 have been the subject of several transgenic overexpression and deletion studies. Both isoforms are highly expressed in the heart and are important negative feedback responses of cardiac rhythmicity (Cosson et al., 2019). While both isoforms regulate heart rate (HR) and contractility, AC6 appears more important at baseline cardiac function (Vatner et al., 2013). AC5 and AC6 may function distinctly in the pathogenesis of cardiac stress responses. The protein abundance of AC5 and AC6 responds oppositely to pressure overload LV hypertrophy (Okumura et al., 2003b; Sugano et al., 2011), with upregulation of AC5 and downregulation of AC6, implying that it is AC5 that plays a role in chronic pressure overload cardiomyopathy (Vatner et al., 2013). It is probable that these two isoforms also play different physiological roles in regulating cardiac function during dilated cardiomyopathy. Both AC5 and AC6 mRNAs appear decreased in dogs with pacing-induced Congestive Heart Failure (CHF) (Ishikawa et al., 1994); while in a swine model of tachypacing and severe heart failure, AC6 but not AC5 is downregulated (Ping et al., 1997). While some studies suggest AC5 overexpression improves cardiac function in transgenic mice during exercise (Esposito et al., 2008), deletion of AC5 protects the heart in most models of cardiac stress including heart failure; in contrast, AC6 overexpression has been correlated with cardioprotective effects (Wu et al., 2017; Ostrom et al., 2022). The overexpression of AC6 leads to increased LV function, improved cAMP and Ca^2+^ handling, and may protect the heart from pressure overload-induced systolic and diastolic dysfunction (Phan et al., 2007; Guellich et al., 2010). AC5 deletion, on the other hand, results in protecting the heart from cardiomyopathy, chronic catecholamine stress, and chronic pressure overload (Okumura et al., 2003b; Okumura et al., 2007). Isoform-specific effects are not restricted to myocardial AC5 and AC6. Cardiac-specific overexpression of AC8 in 3-month-old transgenic mice alters several pathways, resulting in elevated AC activity and cAMP-induced cardiac workload until up to 1 year of age, without excess mortality or onset of heart failure (Tarasov et al., 2022; Qu et al., 2024). However, other studies have shown cardiac overexpression of AC8 to cause early and accelerated cardiac remodeling, resulting in development of heart failure and a shortened life span, suggesting that alterations in cAMP/PKA signaling can hasten cardiac aging, in part via the glycogen-synthase-kinase 3α/β (GSK3α/β) phosphorylation pathway (Mougenot et al., 2019).

**FIGURE 2:**
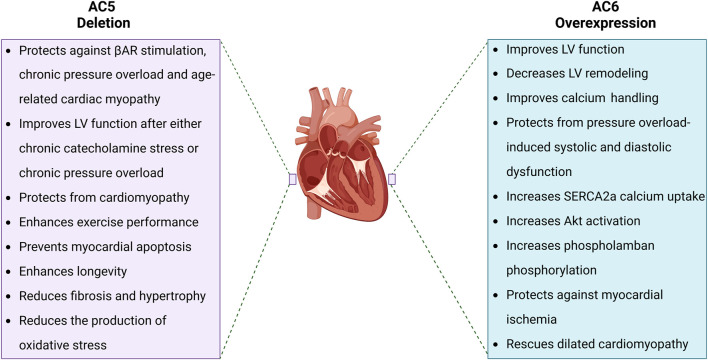
Pathophysiological effects of AC5 deletion and AC6 overexpression. AC5 and AC6 behave distinctly when increased or disrupted. While AC5 deletion has come with several beneficial effects for the heart such as prolonged longevity and enhanced LV function, AC6 overexpression improves cardiac cAMP generation and Ca^2+^ handling, resulting in improved LV function.

Although there remains some controversy regarding the specific regulation and roles of AC5 and AC6 isoforms in controlling cardiac function, their roles in the production of required cAMP to initiate cardiac chronotropic and inotropic responses are indispensable. It is thus important to differentiate the regulation and biological functions of AC5 *versus* AC6, leading the way to development of targeted treatments for different cardiac pathologies. The respective therapeutic outcomes of AC5 deletion and AC6 overexpression in the cardiovascular system are included in Figure 2.

### Regulation of AC5 and AC6 by calcium

Ca^2+^ serves as a pivotal regulator of AC5 and AC6, playing a crucial role in the control of cellular homeostasis and heart function regulation. The inhibition of ACs 5 and 6 by Ca^2+^, which is non-competitive with respect to ATP (Guillou et al., 1999), contributes to pacemaking in cardiac tissue and also sustains endothelial cell permeability. Inhibition of these cardiac AC isoforms by Ca^2+^, which their own cAMP generation permits to enter the cardiomyocyte, serves as a form of feedback control regulating pacemaker rhythmicity as well as the force of contraction (Mou et al., 2009). A wide range of *in vitro* inhibitory sensitivities are reported. Systolic [Ca^2+^] 1 µM is required to inhibit the activity of AC5/6, while at higher intracellular [Ca^2+^] (10–25 µM), the activity of all AC isoforms is inhibited (Guillou et al., 1999; Halls and Cooper, 2017). However, AC5 and AC6 inhibition by Ca^2+^ is biphasic, involving low- and high-affinity binding sites, so Ca^2+^ can inhibit AC5 and AC6 in sub- to supramicromolar concentration ranges (Steer and Levitzki, 1975; HANSKI et al., 1977; OLDHAM et al., 1984; Fagan et al., 1998; Guillou et al., 1999). The sub-micromolar [Ca^2+^] (0.2–0.6 µM) inhibits AC5 and AC6 in membrane preparations from different tissues, cultured cell lines, and in recombinant systems (Beazely and Watts, 2006). Mechanistically, Ca^2+^ antagonizes the activation of AC5 and AC6 by Mg^2+^ (Mou et al., 2009). Site-directed mutations of AC5 in the presence of other divalent cations indicate that the Mg^2+^-binding loci at the AC5 catalytic site are essential for its Ca^2+^-mediated inhibition (Hu et al., 2002; Mou et al., 2009). In addition to being a hub for GPCR AC interactions, lipid rafts are locations for capacitive Ca^2+^ entry channels; *Group III* AC isoforms that are localized in lipid rafts are also regulated by Ca^2+^ (Ostrom and Insel, 2004). Disruption of lipid rafts results in loss of capacitive Ca^2+^ entry and dysregulation of Ca^2+^-regulated AC isoforms (Dessauer et al., 2017).

## Subcellular localization of AC5 and AC6

**FIGURE 3:**
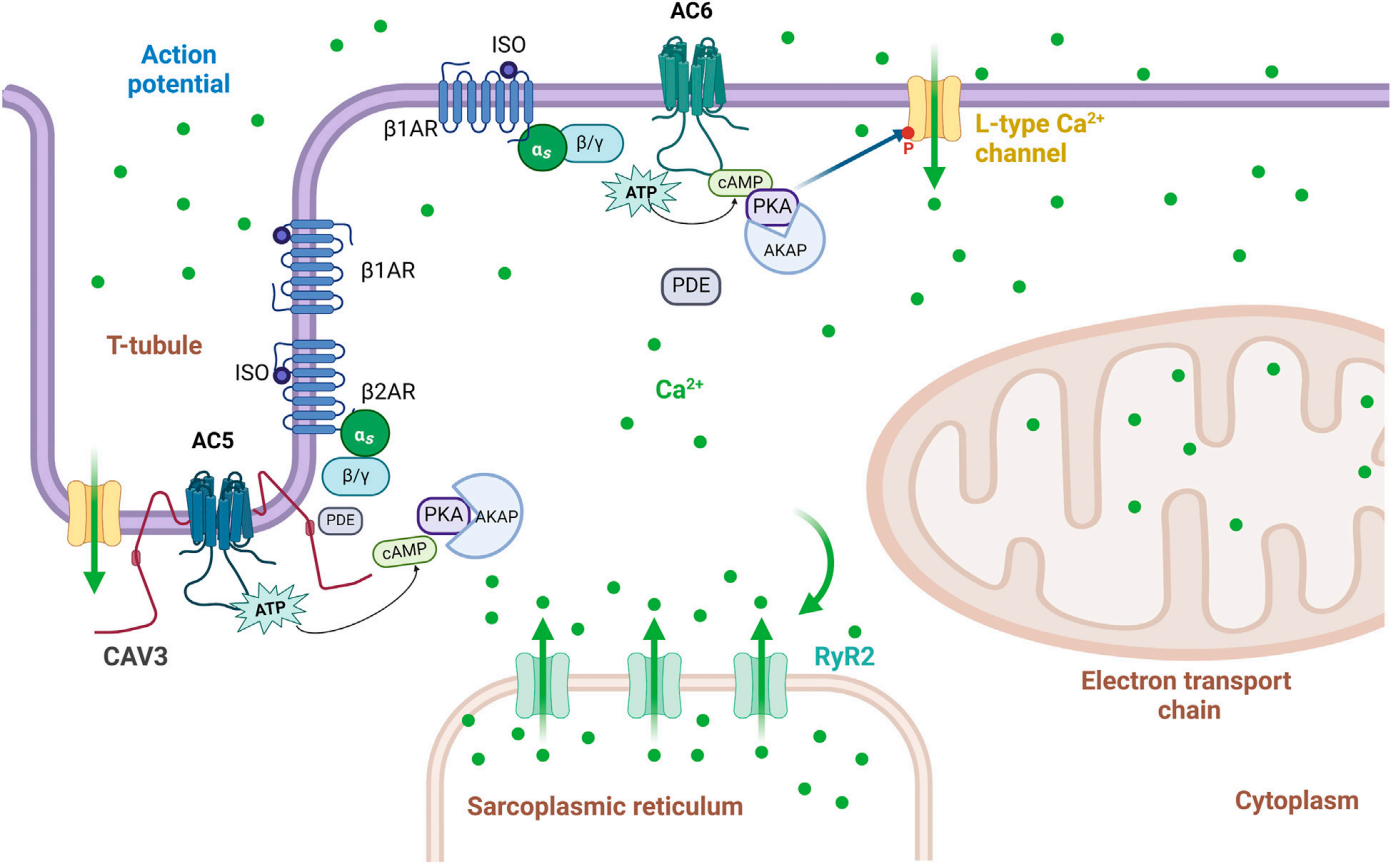
Schematic representation of the localization of AC5 and AC6 signaling within the t-tubule and sarcolemmal membrane. In ventricular myocytes, AC6 is localized to the plasma membrane outside of the t-tubular region, and it interacts only with β1AR signaling-mediated augmentation of the L-type Ca^2+^ current (ICa,L), while AC5 is localized to the membrane in t-tubular regions, and its function on ICa,L is restrained by cAMP degradation by phosphodiesterases. Adapted from (Timofeyev et al., 2013); image designed using BioRender.

GPCR signaling pathways are type-specific for either AC5 or AC6, as determined by the colocalization of receptors and AC isoforms in lipid raft or non-raft plasma membranes (Ostrom et al., 2000). ACs 4–7 are expressed in the heart and vascular system, but among them, AC5 and AC6 are dominant subtypes in adult mammalian heart (Dessauer et al., 2017). The compartmentation of AC5 and AC6 in caveolae or membrane lipid rafts provides a key biochemical process for temporal and spatial segregation of signal transduction as well as cross-talk between signaling cascades, resulting in the compartmentalization of cAMP signaling (Thangavel et al., 2009). While native AC5/6 are found in caveolar fractions, overexpressed AC6 in rat aortic smooth muscle cells is found in non-caveolin-rich fractions, where it is functional and increases cAMP (Ostrom et al., 2002). In these cells, low levels of AC6 overexpression do not much change cAMP levels at baseline nor responses to adenosine A2b receptor challenge (Ostrom et al., 2002; Thangavel et al., 2009). In contrast, in cardiomyocytes, overexpressed AC6 localizes similarly to endogenous AC6, which is targeted to caveolae, demonstrating that GPCR and AC5/6 compartmentation to caveolin-rich membranes is cell-type dependent (Ostrom et al., 2000; Ostrom et al., 2001; Ostrom et al., 2002). Overexpression of AC6 improves βAR responses without affecting signaling of other Gαs-coupled receptors in a variety of cells, including airway smooth muscle, lung fibroblasts, and neonatal myocytes (Sunahara et al., 1996; Liu et al., 2008; Sadana and Dessauer, 2009; Dessauer et al., 2017). In vascular smooth muscle cells (VSMCs), AC3, AC5, and AC6 are the most abundant isoforms in βAR-mediated signaling, but during vasodilation, AC6 is the principal isoform involved in βAR-mediated cAMP/PKA signaling and activation of the ATP-sensitive potassium current, thus playing an important role in setting the membrane potential. Its counterpart AC5 does not have similar activity, (Nelson et al., 2011). In ventricular myocytes, isoforms AC5 and AC6 have separate subcellular compartmentalization. While AC5 is mostly found deep in transverse tubules interacting with caveolin-3 (CAV3) and phosphodiesterases (PDEs), AC6 is located in the plasma membrane outside the t-tubular area (Figure 3) (Timofeyev et al., 2013). βAR receptors are also differentially distributed; β1- and β2AR are found within t-tubules, but β1AR (comprising >70% of cardiac βAR) is found on the sarcolemma outside t-tubules (Brodde, 1991). β1AR triggers cAMP generation, which increases PKA-mediated phosphorylation of L-type Ca^2+^ channels as well as other regulatory proteins, greatly increasing cardiac contractility; β2AR couples with both Gαs and Gαi, resulting in a smaller increment of contractility (Zheng et al., 2005). Inactivation of Gαi by pertussis toxin enhances β1AR-mediated inotropy despite β1AR not coupling to Gαi, indicating that Gαi also inhibits the AC5/6 receptor independently (Cosson et al., 2019). Due to subcellular localization, AC5 has been connected to β2AR signaling, while AC6 is responsible for extra-tubular β1AR signaling as well as β1AR-mediated augmentation of the L-type Ca^2+^ channel current (*I*_Ca,L_) in ventricular myocytes (Timofeyev et al., 2013) (Figure 3). Both βAR subtypes have obvious inotropic effects; dobutamine, a non-selective βAR agonist, increases cardiac contractility in both AC5KO and AC6KO mice (Tang et al., 2006; Tang et al., 2008). While β2AR overexpression enhances ventricular function and activates cell survival pathways (Milano et al., 1994), β1AR overexpression appears catastrophic, causing both cardiac hypertrophy and dilated cardiomyopathy (Engelhardt et al., 1999). The opposite effects have been reported following manipulation of their more proximal AC subtypes; AC5 disruption in t-tubules is cardioprotective (Okumura et al., 2003b), while overexpression of AC6 rescues heart function in cardiac injury (Gao et al., 2016). However, functional coupling may not only reflect localization. In AC6KO, a shift from dominant AC6 coupling to AC5 coupling occurs in the β1AR signaling cascade. This change in the AC assignment in AC6KO results in rearranged signaling compartmentalization as well as an alteration in the PDE isoform control of the cAMP pool (Cosson et al., 2019; Ostrom et al., 2022). These findings suggest some functional redundancy of AC5 or AC6 for β1AR-mediated inotropic responses. Additionally, in CHF models largely ascribed to excess β1AR activity, β2AR redistributes to the cell surface, and this loss of cAMP compartmentation correlates with heart failure (Nikolaev et al., 2010). Studies of detubulated cardiomyocytes reveal that stimulation of both β1AR and β2AR is more functionally effective at the sarcolemmal surface, rather than within t-tubules (Cros and Brette, 2013). Pharmacological inhibition studies confirm that β1AR preferentially couples with AC5 at t-tubules, while β2AR couples with AC6 when present at the cell surface, resulting in the observed AC subtype-specific effects on cardiac function and survival signaling (Tsunematsu et al., 2015).

## Compartmentalization of cAMP signaling in the heart

**FIGURE 4:**
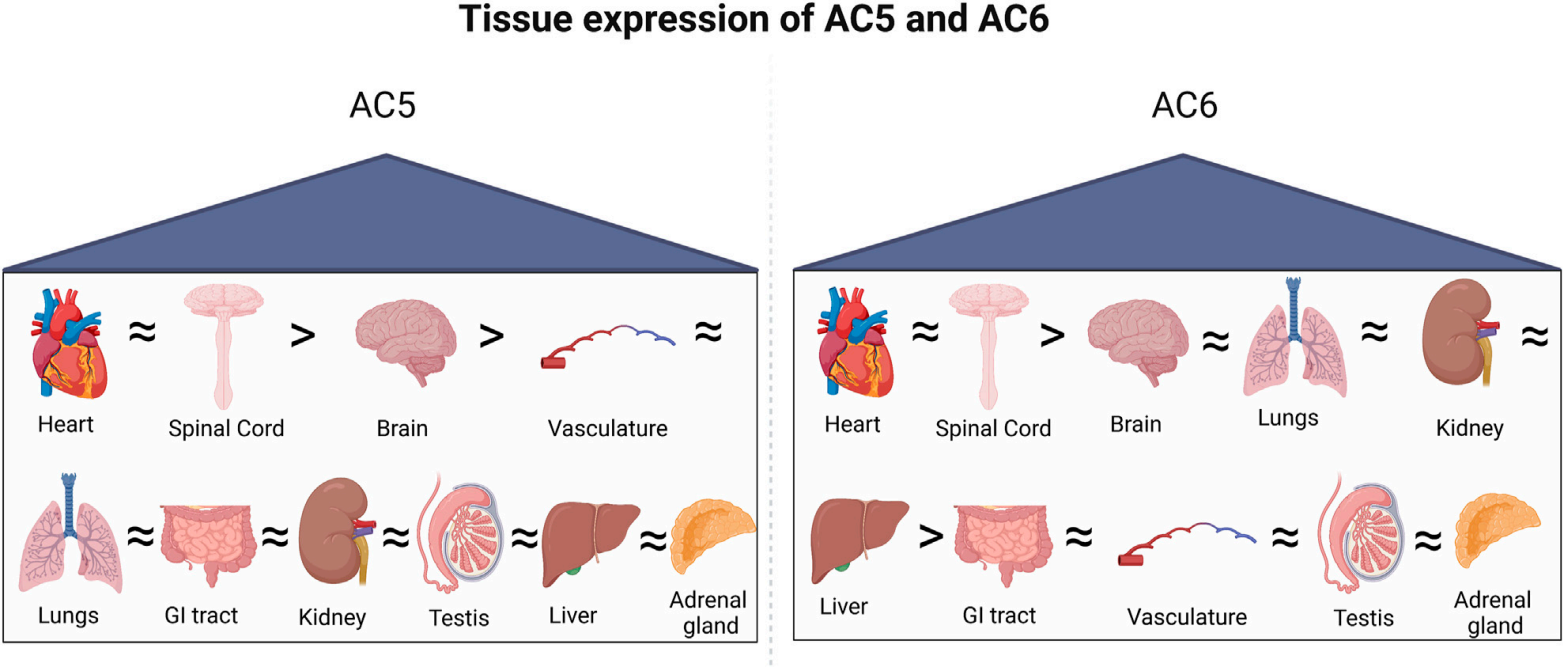
Tissue-specific expression of AC5 and AC6. Both isoforms are highly expressed in the heart and spinal cord, followed by the brain.

While most tissues express a plethora of AC isoforms (Figure 4), the specific roles of each isoform may overlap within that cell type (Sadana and Dessauer, 2009). Functional redundancy or compensatory roles have been proposed within individual AC groups, as they are regulated or expressed in a comparable manner (Defer et al., 2000). Changes in AC activity may depend on the profile of isoforms present in a certain cell or tissue (Tao et al., 1998). Differing phenotypes of AC5 and AC6 activity suggest the maintenance of distinct pools of cAMP within cardiac myocytes, generated by each enzyme. Regulation and localization of AC5 and AC6 isoforms tailor the generation of cAMP by concurrent signals, assisting in dynamic control of the cAMP signal. The compartmentation of cAMP signaling in the heart arises, in part, from specific signaling complexes which generate distinct cell responses, with AC5 and AC6 being central effectors to the formation and maintenance of these compartments.

The organization of cAMP microdomains in isolated cardiac cells has been researched extensively, using various effector proteins to sort distinct cAMP pools (Patel and Gold, 2015). Several downstream effectors can regulate cAMP production or hydrolysis, including PDEs, PKA, cyclic nucleotide-gated (CNG) channels, hyperpolarization-activated cyclic nucleotide-gated exchange proteins, exchange protein directly activated by cAMP (Epac), and the Popeye domain-containing (POPDC) protein family. At the level of cAMP signal longevity, PDE isoforms, importantly PDE3/4, play pivotal roles in the discrete regulation of localized cAMP levels in neonatal cardiac myocytes (Willoughby and Cooper, 2007). PDE4 isoforms regulate cAMP signaling resulting from β1AR and β2AR stimulation in cardiac myocytes; PDE4B controls β1AR but not β2AR responses in cardiac myocytes, suggesting a localized function to control β1AR-dependent excitation–contraction coupling (Mika et al., 2014). PDE3 acts globally on cAMP in normal and heart failure models, modulating cAMP-mediated regulation of Ca^2+^ re-uptake in the sarcoplasmic reticulum (SR) (Calamera et al., 2022). Spatial and temporal organization of cAMP-dependent pathways is further provided by cAMP-related protein scaffolding, biasing the cAMP signal toward different end functions.

### AKAP organizes AC5 and AC6 cAMP in the heart

A-kinase-anchoring proteins (AKAPs) are scaffold proteins which coordinate signaling components into multiprotein complexes, giving rise to regulation of the spatial and temporal organization and fine-tuning of cAMP signaling (Kritzer et al., 2012), ensuring correct targeting of cAMP-dependent PKA and other signaling enzymes to precise subcellular compartments (Diviani et al., 2016). AKAPs function through binding to the regulatory subunits of PKA and crucial PKA phosphorylation sites to initiate rapid and targeted coupling of the kinase to downstream effectors (Ostrom et al., 2022). AC5/6 in the heart utilizes the interactions with AKAPs to cohort with upstream and/or downstream effectors. Over 50 AKAPs are known to target PKA to various cellular locations; important cardiovascular AKAPs include AKAP15/18, AKAP79/150, Yotiao, mAKAP, AKAP-Lbc, and Gravin (Baldwin and Dessauer, 2018). The interaction of these AKAPs with AC5 or AC6 determines the localization and activity of their cAMP signaling complexes (Efendiev and Dessauer, 2011). As scaffolding effectors, AKAPs not only anchor the localization of ACs but also preserve local pools of cAMP by assembling macromolecular complexes (Baldwin and Dessauer, 2018). In cardiomyocytes, the arrangement of MAP kinase, Ca^2+^, and cAMP-dependent pathways is coordinated through the muscle A-kinase anchoring protein β, playing a key role in cellular hypertrophy (Kapiloff et al., 2009). AC5 binds to a specific N-terminal site on mAKAP-(245–340), resulting in impairment of AC5 activity; interruption of mAKAPβ–AC5 complexes could be targeted therapeutically to reduce cAMP generation and hypertrophy in cardiac myocytes (Bauman et al., 2006; Kapiloff et al., 2009). Both AC5 and AC6 interact with AKAP79/150 along with PKA, generating a negative feedback loop where cAMP production is impaired by PKA phosphorylation of AC5/6 (Baldwin and Dessauer, 2018).

### AC5 and AC6 cAMP regulation by Epac in the heart

Another downstream cAMP effector is Epac, a family of cAMP-regulated guanine nucleotide exchange factors functioning independently of PKA as mediators of cAMP signaling. While considered less important in cardiomyocytes than PKA, Epac has high affinity to bind to cAMP, resulting in activation of small GTPases Rap1 and Rap2 (Tan et al., 2022). Epac1 and Epac2 play roles in cardiovascular Ca^2+^ signaling and vascular endothelial barrier formation (Lezoualc’h et al., 2016). AC5 transgenic mice with knockout of the Epac1 gene had decreased cardiac dysfunction and were less susceptible to pacing-induced atrial fibrillation after chronic isoproterenol infusion compared to controls while evincing less cardiac apoptosis and fibrosis than the AC5 transgenics, suggesting that Epac1 mediates the deleterious effects of AC5 overexpression on cardiac function and rhythmogenesis (Cai et al., 2016). On the other hand, mice lacking Epac1 are protected against pressure overload or chronic catecholamine stress-induced cardiac dysfunction (Fujita et al., 2017), reinforcing the notion that rate- and pressure-induced cardiac failure may arise from separate cAMP pools.

## Advances in cAMP biosensors in live cells

Various constructs have been developed to measure real-time cAMP levels in living cells or tissues. Previous biochemical methods or radioligand tools were not able to precisely measure the spatiotemporal resolution of intercellular cAMP fluctuations; however, several fluorescent or luminescent biosensors have been introduced that can track real-time cAMP levels in living cells. These cAMP sensors have the advantage of facilitating the study of complex, compartment-specific cAMP-dependent responses (Warrier et al., 2005).

Classical methods of determining AC activity have involved detection of radiolabeled substrate ATP conversion to AMP and detection of enzyme reaction products by chromatography (Streeto and Reddy, 1967; Krishna et al., 1968); these methods can be applied to cell or tissue lysates. Loss of ATP-bound lanthanide fluorescence has been used as an indirect but specific indicator of AC ATPase activity (Spangler et al., 2008b). The terbium–norfloxacin AC activity assay measures the substrate turnover as an assessment of AC catalytic function; varying the exogenous ATP substrate concentration permits the calculation of Michaelis–Menten kinetics (Spangler et al., 2008a). In contrast, intact cell cAMP assays are cumulative measures of product formation in the absence of exogenous substrates, reflecting real-time steady state inclusive of cAMP generation, degradation, or export. Genetically encoded cAMP sensors can also be targeted to subcellular location.

Fluorescence or Förster resonance energy transfer (FRET) uses an excited donor fluorophore to transfer energy to a proximal acceptor fluorophore, which then emits a photon of a lower energy, resulting in detectable red-shift of the emitted light. FRET biosensors used to detect cAMP include PKA-, Epac-, and CNGC-based cAMP sensors. The original FRET PKA-based cAMP biosensor uses fluorescein (donor) and rhodamine (acceptor) to measure the decrease in the FRET signal due to elevation in intracellular cAMP, leading to dissociation of the regulatory subunit of PKA from its catalytic segment (Adams et al., 1991). This sensor was the first to measure microdomain cAMP levels, demonstrating a striated pattern in neonatal rat ventricular cardiomyocytes because of the interaction of PKA with AKAPs near Z-lines. These sensors have also been used to interrogate the roles of PDE3 and PDE4 in cAMP compartmentation in neonatal rat ventricular myocytes (Mongillo et al., 2004). However, the PKA-based biosensor has some limitations: a low signal-to-noise ratio, narrow dynamic range, slow response time, and need for transfection of two distinct constructs (Ponsioen et al., 2004). Epac-based cAMP biosensors address some of these issues. In these, Epac activation is determined in live cells by sandwiching Epac between cyan fluorescent protein (CFP) and yellow fluorescent protein (YFP), taking advantage of a conformational shift generated by cAMP binding. When using cAMP-lowering agonists, FRET fully recovers, but quickly diminishes in response to cAMP-raising agents. As a result, CFP–Epac–YFP is considered a very sensitive cAMP indicator (Ponsioen et al., 2004). Epac-based cAMP biosensors have been used for monitoring Gi/o-mediated cAMP reduction after stimulation of adrenergic α2A or μ opioid receptors in a time-resolved manner without pre-stimulation with FSK or IBMX to increase endogenous cAMP levels (Storch et al., 2017), indicating detection of low levels of cAMP. The use of FRET-based sensors to visualize cAMP signaling microdomains by fluorescence microscopy has been well-reviewed (Calebiro and Maiellaro, 2014).

**FIGURE 5:**
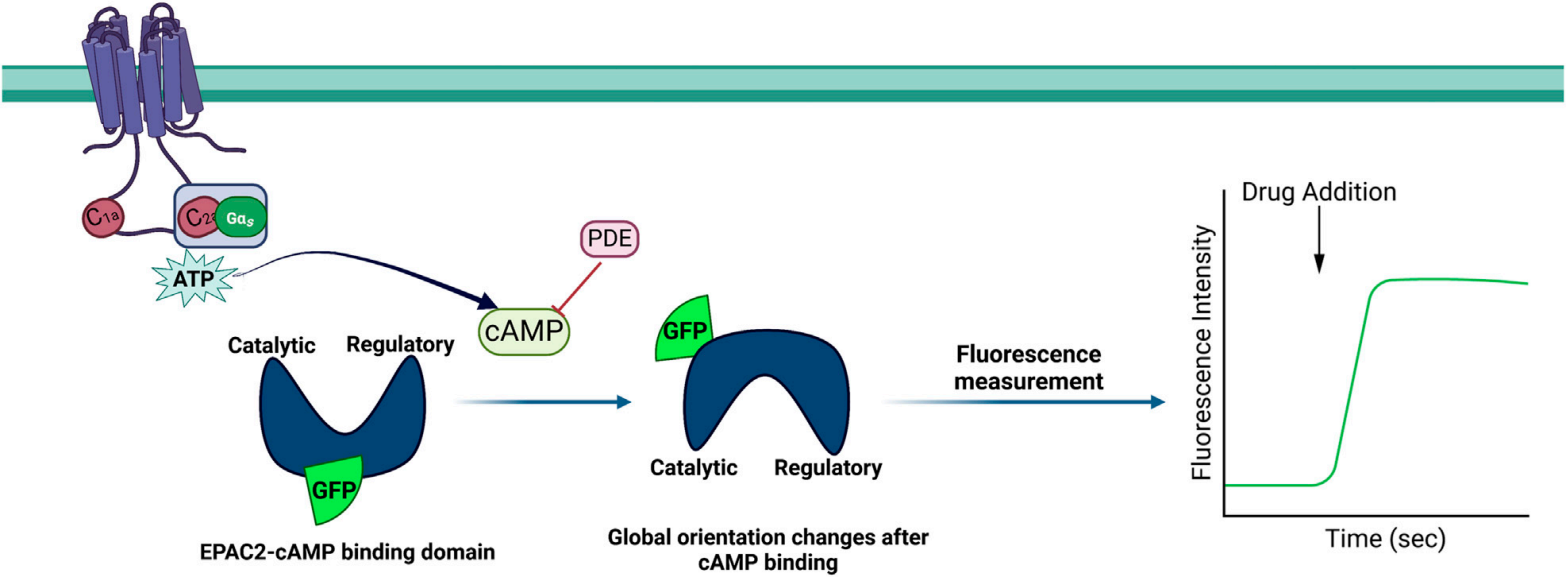
Schematic illustration of the cAMP difference detector *in situ* (cADDis) assay. The enzymatic and catalytic domains of the guanine nucleotide exchange factor Epac2 were joined to form circularly permuted green fluorescent protein (cpGFP). The binding and unbinding of the cAMP molecule cause a conformational change in Epac2.

A newer, versatile approach for measuring cAMP after Gαs- or Gαi-mediated signaling is the cAMP difference detector *in situ* (cADDis) assay (schematic representation, Figure 5). This sensor uses a single fluorescent protein, the Epac cAMP-binding domain fused to circularly permuted mNeonGreen and red mMaple, resulting in a readily detectable bright green fluorescence signal; the Upward cADDis sensor manifests a rise in green fluorescence with increased cAMP, while Downward cADDis decreases fluorescence in response to increased cAMP (Tewson et al., 2018). The cADDis sensor is capable of differentiating basal cAMP from that stimulated by receptor agonism as well as cAMP changes due to Gαs or Gαi activation, when expressed in HEK293T cells (Tewson et al., 2018). Due to its robust expression in multiple cell types and ability to measure real-time cAMP kinetics in live cells over long periods of time with little loss of sensor signals, cADDis has been used in many recent studies of AC cAMP production (Baldwin et al., 2019; Bhatia et al., 2023; Cattani-Cavalieri et al., 2023; Ripoll and Von Zastrow, 2023). Other transfectable single-fluorescent protein cAMP sensors in use include fluorescent cAMP indicator (Flamindo) (Odaka et al., 2014), red fluorescent indicator for cAMP (R-FlincA) (Ohta et al., 2018), and cAMPr (Hackley et al., 2018).

BRET biosensors (bioluminescence resonance energy transfer) use a donor luciferase to oxidize luciferin-generating bioluminescence that excites the acceptor, emitting light at an extended wavelength (Wu and Jiang, 2022). BRET enables detection of fusion protein interactions directly, without the need of an external light source to stimulate a fluorescent energy donor (Prinz et al., 2006). This technology further addresses the problems of photobleaching, autofluorescence, and signal-to-noise ratio in FRET methods; the drawbacks include difficulty of substrate delivery and stoichiometry and potential for cytotoxicity (Wu and Jiang, 2022). BRET-based Epac1-, PKA-, and nano lantern-based cAMP sensors have been developed to investigate cAMP generation (Masri et al., 2008; Hunter et al., 2017; Valkovic et al., 2018).

All plasmid-based cAMP sensors have advantages and disadvantages, with respect to ease of introduction without toxicity, targetability of expression in specific subcellular locations, temporal and spatial resolution of cAMP detection, cAMP detection ranges, and buffering from metabolic processes or post-translational modifications in living cells or tissues. Rapid cAMP binding, instant transmission of signals, and rapid reversibility of the binding event would denote an ideal cAMP sensor (Paramonov et al., 2015). Further optimization is needed for detection of low unstimulated cAMP levels in sensitive primary cells or tissues. Available cAMP biosensors have recently been reviewed in detail (Kim et al., 2021).

## Regulatory post-translational modifications of AC5 and AC6

### AC5/6 phosphorylation by PKA and PKC

**FIGURE 6:**
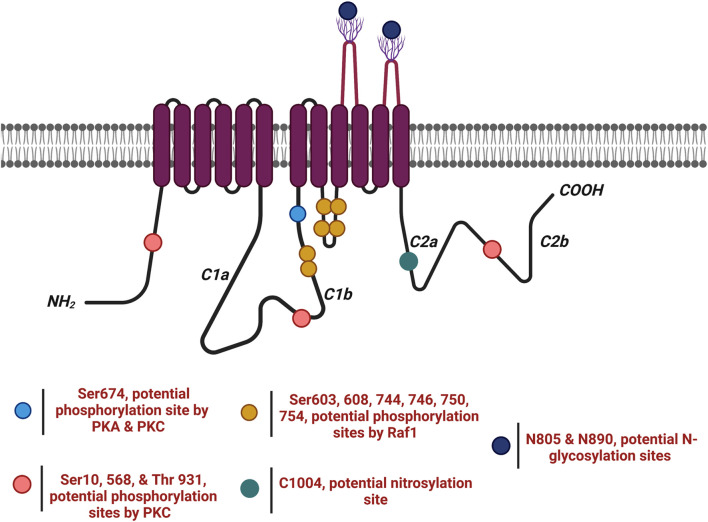
AC6 undergoes several post-translational modifications (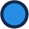). AC6 is phosphorylated by PKA and PKC at Ser674 (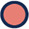); phosphorylated by PKC at Ser10 and 568 and Thr931 (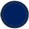); S-nitrosylated by NO at C1004 (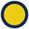); Raf1 on Ser603, 608, 744, 746, 750, and 754 (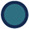); and glycosylated at N805 and N890 on extracellular loops 5 and 6.

PKA and PKC modify AC5 and AC6 activity by phosphorylating serine (Ser) or threonine (Thr) residues. In the heart, PKA regulates metabolism, gene transcription, ion fluxes, and contraction (Colombe and Pidoux, 2021) and also acts as a feedback inhibitor for both AC5 and AC6 through inhibitory phosphorylation near the end of the C1b domain, resulting in the desensitization of AC activity (Sadana and Dessauer, 2009). Stimulation of AC6 was lost after treatment of PKA even in the presence of high concentrations of active Gαs (Chen et al., 1997). Mutational analysis indicates Ser674 is the target of phosphorylation and inhibition of AC6 by PKA in intact cells (Figure 6) (Beazely and Watts, 2006; Chen et al., 1997). AC5 possesses 14 putative PKA phosphorylation sites, including a Ser 788 that corresponds to Ser674 in AC6; however, a specific PKA phosphorylation site in AC5 has not been confirmed (Iwami et al., 1995; Chen et al., 1997).

While both AC5 and AC6 isoforms are inhibited through phosphorylation by PKA, PKC inhibits only AC6 (Kamide et al., 2015; Liu et al., 2022). Previous studies have shown that AC2, AC3, and AC5 can be stimulated by PKC, while AC6 activity is inhibited (Lai et al., 1997; Defer et al., 2000). Phosphorylation of AC5 by PKCα or PKCζ enhances basal activity as well as FSK- or Gαs-stimulated cAMP accumulation; PKC-ζ phosphorylation of AC5 results in a 20-fold increase in AC activity (Kawabe et al., 1996). In the heart, generation of phosphatidyl-inositol-3,4,5 triphosphate via hormonal or growth factor activation of phosphatidyl-inositol 3-kinase can activate PKC-ζ, thus directly activating AC5 production of cAMP (Hanoune and Defer, 2001). In contrast, PKC activators have either no effect or inhibitory effect on AC6 activity (Chen and Iyengar, 1993; Jacobowitz et al., 1993; Lai et al., 1997; Lai et al., 1999). Mutational analysis of the N-terminus of AC6 as a regulatory domain showed that elimination of residues from 1 to 86 or a single mutation of Ser10 prevents phosphorylation and inhibition of AC6 by PKC (Figure 6) (Lai et al., 1999). Subsequent studies also identified AC6 C1 Ser 568 and 674 as well as AC6 C2 Thr931 as inhibitory targets of PKC, suggesting that phosphorylation of this complex of Ser and Thr might trigger a conformational change in the catalytic core, changing AC6 catalytic activity (Lin et al., 2002).

### AC5/6 regulation by glycosylation

AC6 can be glycosylated on two asparagine residues, namely, N805 and N890, on extracellular loops of TM2 (Figure 6) (Wu et al., 2001). The glycosylation of AC6 results in alteration of not only its catalytic activity but also its regulation by Gαi or by PKC. Inhibition of glycosylation by tunicamycin impairs FSK-stimulated AC6 activity, and mutation of glycosylation sites resulted in significantly lower FSK-, Mn^2+^-, and Mg^2+^-stimulated enzyme activities than did wild-type AC6, suggesting that glycosylation may be necessary for maintenance of AC6 activity (Wu et al., 2001). AC5 glycosylation has not been verified.

### AC5/6 regulation by nitrosylation

Cardiac function, airway, and vascular tone, as well as regulation of immunological defense and neuronal plasticity, are regulated through nitric oxide or reactive nitrogen species (Bhatia et al., 2021). NO-mediated S-nitrosylation of AC5/AC6 plays a crucial regulatory role in physiological processes of cardiovascular function. Previous studies showed that NO, independent of its action on the guanylyl cyclase (GC) pathway, inhibits cAMP in *Dictyostelium discoideum*, indicating that NO can have a direct regulatory effect on ACs via modification of an AC regulatory domain (McVey et al., 1999). Agonist-stimulated cAMP accumulation is inhibited when N18TG2 neuroblastoma cells (Tao et al., 1998) or cardiac myocytes (Joe et al., 1998; Vila-Petroff et al., 1999) are treated with NO or NO donors (Tao et al., 1998). This inhibition is not dependent on the effect of Gαi (McVey et al., 1999) nor on PDE activity (Watson et al., 2001). NO or NO donors were shown to selectively decrease FSK-stimulated AC5/6 activity but not AC1 or AC2 (Hill et al., 2000), while calmodulin stimulation of AC1 is inhibited by NO (Duhe et al., 1994). NO suppression of hormonal or FSK-stimulated AC activity in neuroblastoma plasma membranes does not require CaM (McVey et al., 1999) and directly inhibits FSK-stimulated AC5 and AC6 activity (Hill et al., 2000).

The presence of caveolae, coincidentally the location of endogenous NO generation by endothelial nitric oxide synthase (eNOS), is required for NO inhibition of AC6 activity. Inhibition of AC5/6 by NO relies upon their localization in lipid rafts with caveolin signaling complexes (Ostrom et al., 2004). While lipid raft depletion with β-cyclodextrin prevented the activation of AC activity by βAR and Gαs, it has no influence on the prostanoid receptors, which are located outside of caveolin-rich microdomains and can still activate AC. Both native cardiac myocytes and pulmonary artery endothelial cells overexpressing AC6 are inhibited by the NO donor S-nitroso-N-acetylpenicillamine (SNAP), inhibiting both basal and FSK-stimulated cAMP production (Ostrom et al., 2004). This process is subject to reversal by reducing agents, indicating the involvement of cysteine residue(s) as the target for S-nitrosylation (McVey et al., 1999; Ostrom et al., 2004). The reaction between NO and superoxide (O_2_^−^) results in the production of reactive nitrogen oxygen species (RNOS) capable of altering a broader variety of biomolecules than NO itself (Ridnour et al., 2004). At higher levels of O_2_^−^, NO inactivation is followed in turn by generation of the potent and short-lived oxidant peroxynitrite (ONOO^−^/ONOOH), which can directly react with metal ions and thiols (Piacenza et al., 2022). ONOO^−^ contributes more to S-nitrosylation of adjacent proteins than does NO (Maccarrone et al., 2000). It is also proposed that inhibition of AC by NO may be through S-nitrosylation caused by the reaction of another NO intermediate, nitrosonium (NO+), with cysteine residues (Tao et al., 1998). In a quantitative mass spectrometry screening investigation of modified cysteines utilizing a bioorthogonal cleavable-linker switch technique, AC6 featured among proteins identified as S-nitrosylated (Mnatsakanyan et al., 2019). AC6 inhibition due to S-nitrosylation was also demonstrated in pulmonary arteries (Sikarwar et al., 2018). Identification of AC6 cysteines susceptible to S-nitrosylation has been explored using site-directed mutagenesis of cysteines identified by bioinformatics analysis to reside within an SNO motif (Jaggupilli et al., 2018; Bhagirath et al., 2022). Mutation of cysteine 1004, located in the conserved C2 domain of AC6 near the Gαs docking position (Figure 6), decreases the basal and stimulated activity of AC6, indicating the importance of this residue in AC6 for intact catalytic activity and also susceptibility to inhibition if nitrosylated (Bhagirath et al., 2022).

### AC5/6 regulation by other PTMs

AC6 may be phosphorylated through receptor tyrosine kinases (RTKs); Ser residues 603, 608, 744, 746, 750, and 754 have been implicated following RTK activation by IGF-1 or tyrosine phosphatase inhibition with sodium orthovanadate (Figure 6). Augmentation of AC6 catalytic function following RTK activity is inhibited by endogenous p74^raf−1^ activity, but not by inhibitors of ERK, PKC, PKA, or PI3 kinase activity (Tan et al., 2001).

## Role of AC5 in cardiovascular function

In this section, we review the studies reporting the role of AC5 in cardiomyopathies induced by catecholamine stress including chronic isoproterenol stimulation, aging, and pressure overload. Upregulated AC5 mRNA expression in spontaneously hypertensive rats suggests complex regulation of LV hypertrophy in hypertension (Vatner et al., 2013). Myocardial AC5 mRNA increases from 5 to 12 weeks in spontaneously hypertensive rats, associated with the development of LV hypertrophy (Fujino et al., 2003). On the other hand, AC5KO mice have diminished sympathetic and parasympathetic responses and disrupted Ca^2+^-mediated cardiac regulation. Both basal and isoproterenol-stimulated AC activity are attenuated at 30%–40% in the cardiac membranes of AC5KO mice, with no compensatory increase in other AC isoforms; this reduction in AC activity does not alter cardiac function at baseline but compromises the LV inotropic response to adrenergic stimulation (Okumura et al., 2003a). In pressure overload, while there was no difference between AC5WT and AC5KO in cardiac muscle mass at baseline, AC5KO protected the heart from deleterious effects of pressure overload on LV ejection fraction, through restriction of myocardial apoptosis via upregulation of BCl-2 (Okumura et al., 2003b). Others have demonstrated enhancement in basal LV function in AC5KO as well as impairment in the responsiveness of LV to βAR stimulation (Tang et al., 2006). AC5KO mice also showed better heart function following chronic catecholamine stimulation, again through reduction in cardiac apoptosis, due to increased Bcl-2 expression and Akt signaling (Okumura et al., 2007). In contrast to the outcomes after prolonged isoproterenol infusion in AC5WT and AC5KO mice, the ejection fraction response of the LV to an abrupt isoproterenol challenge was lowered in AC5KO, which is in line with the downregulation of AC5 catalytic activity. These data indicate that AC5 impairs survival signaling after long-term catecholamine infusion, while AC5 deletion improves cardiac desensitization, suggesting a novel strategy for heart failure therapy.

Zhang et al. investigated ways in which selected Gαs-coupled receptors (GsPCRs) regulate cardiomyocyte viability by generating distinct signaling complexes governing pro-survival *versus* pro-death signaling. It was revealed that among the five GsPCRs studied, stimulation of β1AR and histamine-H2-receptor (H2R) has negative effects on the survival of cardiomyocytes, mediated through cAMP production by AC5 but not AC6 and via PKA activity stimulating pannexin-1 to release ATP into the extracellular space. On the other hand, activation of pro-survival GsPCRs adenosine-A2-receptor (A2R), calcitonin-gene-related-peptide-receptor (CGRPR), or relaxin-family peptide-receptor 1 (RXFP1) results in protective effects on cardiomyocyte survival as a function of cAMP generation by AC6, resulting in activation of cAMP efflux pumps. These findings indicate that selection of AC5 *versus* AC6 by GsPCRs determines cAMP localization which controls cAMP fate, thus altering cardiomyocyte survival (Zhang et al., 2023).

In an examination of heart rate variability during transient microgravity in parabolic flight, autonomic dysregulation became worse in AC5KO mice, while heart rate stability improved as a function of AC5 overexpression, indicating that AC5 may improve autonomic regulation (Okumura et al., 2008). It was further shown that AC5 activity is required to achieve constant responses in the low- and high-frequency ratio or normalized high frequency, two markers of sympathetic and parasympathetic activity, respectively (Bai et al., 2012). Thus, while inhibition of AC5 is beneficial for preventing cardiac myocyte myocardial apoptosis induced by excessive βAR stimulation, activation of this isoform may be advantageous in acute heart failure with low rate.

Transgenic mice overexpressing both AC5 and Gαq show efficient βAR-stimulated AC activity and cardiac contractility, but their hearts also appear pathologically fibrotic and hypertrophic (Tepe and Liggett, 1999). Microarray analysis of these hearts showed upregulation of various genes involved in pressure overload LV hypertrophy (Park et al., 2011). Examining genes relevant to ventricular hypertrophy upregulated in AC5 transgenic hearts even at baseline, transcription factor binding analysis revealed enrichment for the binding sites of nuclear factor of activated T-cells (NFAT), vital for the development and progress of cardiomyocyte hypertrophy (Park et al., 2011). NFAT binding determines the expression of cytoskeletal proteins (Schubert et al., 2003). So AC5 overexpression can mediate calcineurin–NFAT signaling involved in the development of LV hypertrophy (Park et al., 2011).

AC5KO mice also demonstrate increased physical performance, mediated through upregulation of the sirtuin-1 (SIRT1) pathway and regulating the antioxidant enzyme manganese superoxide dismutase (MnSOD) in the heart and liver of ACKO mice (Yan et al., 2012). It is proposed that SIRT1 is inhibited by AC5, leading to disruption of an interaction between SIRT1 and forkhead box O3 (FoxO3a), which eventually decreases MnSOD expression, thereby augmenting oxidative stress. The increase in MnSOD in myocytes subject to adenoviral AC5 KO is abolished by inhibition of either MEK or sirtuin, indicating MnSOD upregulation by both the mitogen-activated protein kinase kinase/extracellular signal-regulated kinases (MEK/ERK) and SIRT1/FoxO3a pathways (Chester and Watts, 2007; Lai et al., 2013). AC5KO mice are found to be resistant to cardiac stress with an enhanced median life span of roughly 30%, with protection from bone demineralization, and decreased susceptibility to fractures or aging-induced cardiomyopathy (Yan et al., 2007). The longevity, healthful aging, and stress resistance detected in AC5KO mice were correlated to diminished cAMP and PKA, resulting in activation of the Raf/MEK/ERK pathway, leading to the enhanced level of MnSOD (Yan et al., 2007). Additionally, AC5KO appears to increase NO signaling, demonstrating another mechanism by which AC5 may antagonize beneficial pathways induced by exercise (Guers et al., 2017). Two highly expressed genes encoding glutathione S-transferase (Gstk1 and Gstm) were downregulated in hypertrophic hearts, importantly because glutathione S-transferase plays the role of an antioxidant by conjugating glutathione on various substrates to protect the heart from oxidative stress (Cho et al., 2003; Yan et al., 2012).

**FIGURE 7:**
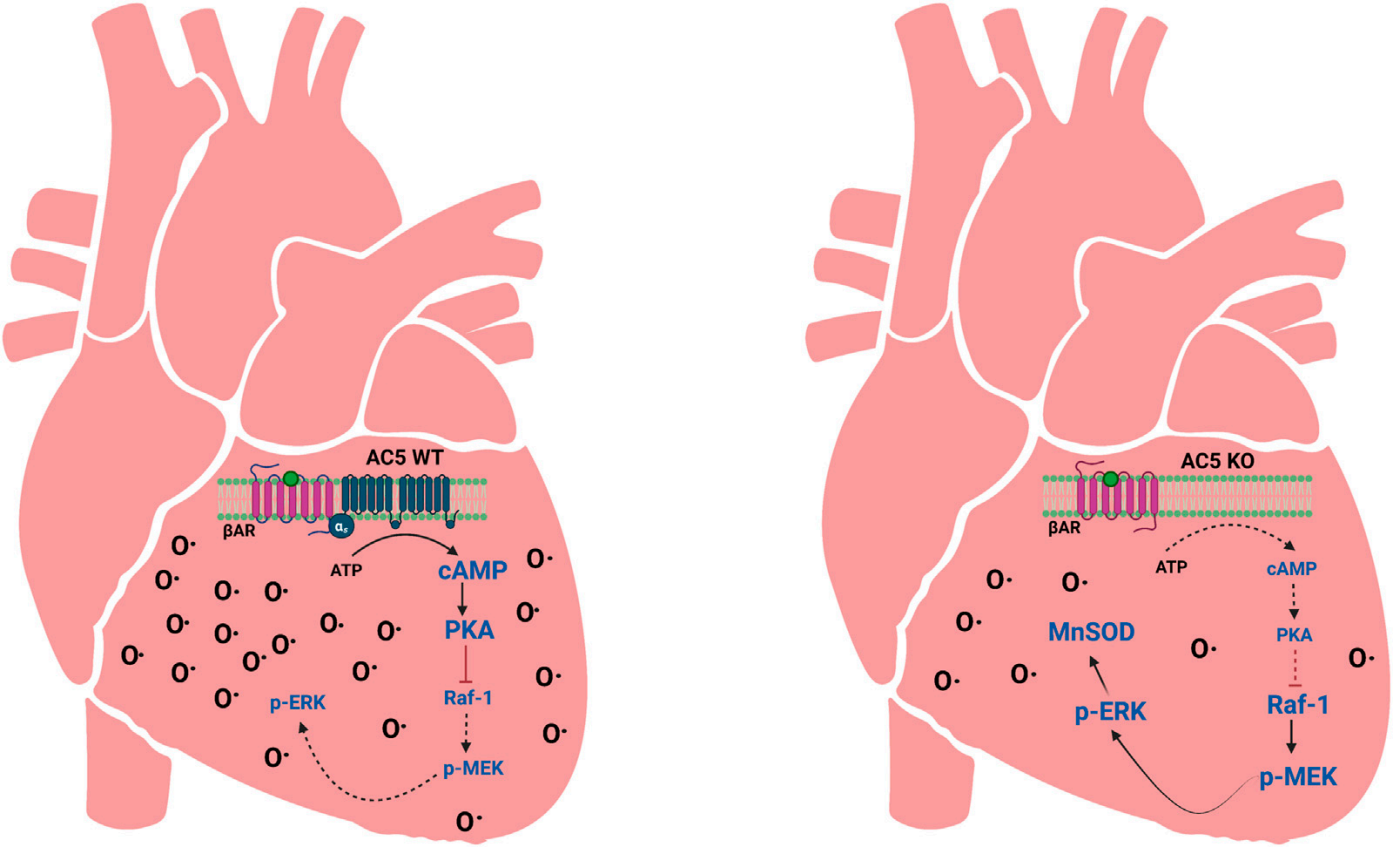
Depiction of AC5 signaling effects on cellular antioxidant imbalances in AC5WT and AC5KO hearts. When AC5 is disrupted (AC5KO), there is less βAR-stimulated cAMP, which decreases PKA activity. Lack of PKA leads to the activation of the Raf-1/MEK-ERK signaling pathway to increase the expression of MnSOD. This results in attenuation of oxidative stress. MnSOD antioxidant defense is not as present in AC5WT. O• indicates oxidative stress. Adapted from (Chester and Watts, 2007); image designed using BioRender.

Overall, these data place AC5 in a crucial role regulating life span and cardiac stress resistance. Figure 7 summarizes AC5 signaling in the context of cellular antioxidant imbalances in AC5WT and AC5KO. Studies delineating the role of AC5 in cardiovascular function are highlighted in Table 1.

**TABLE 1:** Physiological and pathophysiological effects of AC5 and AC6.

Study of cardiomyopathy	Physiological and pathophysiological effects			
	AC5	Ref	AC6	Ref
Chronic pressure-overload	AC5^−/−^ showed better toleration, increased LV function, and less fibrosis and protected from apoptosis	Okumura et al. (2003b)	AC6-Tg (1) increased LV systolic wall stress, (2) reduced LVEF, and (3) unpreserved cardiac function	Guellich et al. (2010)
	AC5-Tg (1) manifested various upregulated genes related to LV hypertrophy and (2) marked enrichment of NFAT binding sites	Park et al. (2011)	Activation of AC6 expression led to better Ca^2+^ handling due to enhanced PLN phosphorylation, decreased NCX1 and PP1 expression, and increased SR [Ca^2+^]	Sugano et al. (2011)
	Expression of AC5 but not AC6 is upregulated in AMPKα2 KO-TAC mice, leading to cause hypertrophy and aggravating of cardiac function, as evidenced by a higher decrease in EF	Garnier et al. (2023)	AC6KO resulted in reduced LV hypertrophy with no sign of LV dilation and protection of LV function in female mice. This was further correlated with reduced expression of FHL1 and periostin	Tang et al. (2010)
Longevity	AC5KO did not change in exercise performance. Increase in exercise capacity in AC5KO mice results in enhanced skeletal muscle function, not cardiac function	Vatner et al. (2015)	Cardiac-directed expression of AC6 protects the heart from myocardial hypertrophy, improves cardiac function, increases cAMP generation, and prolongs survival in Gαq cardiomyopathy	Roth et al. (2002)
Chronic catecholamine stress	MnSOD expression was downregulated, and its effect was 40% lower in AC5-Tg than in WT, but 40% higher in AC5KO, leading to cause chronic catecholamine stress	Lai et al. (2009)	AC6 ^ΔN/ΔN^ and AC6^−/−^ mice (1) responded more to apoptotic myocytes and cardiac remodeling. (2) AC6 plays the main role on Src-dependent STAT3 activation in the sarcolemma and preserved cardiomyocytes against cardiac stress through a PKA/STAT3-dependent pathway	Wu et al. (2017)

## Role of AC6 in cardiovascular function

In contrast to AC5, several studies have shown that increase in AC6 expression is beneficial for the failing heart, preserving LV contractile function and reducing dilation and dysfunction in hearts showing pressure overload (Takahashi et al., 2006; Sugano et al., 2011). The protective effect of AC6 in the heart is postulated as cAMP pathway dependence and independence (Tang et al., 2004; Gao et al., 2008; Gao et al., 2009; Gao et al., 2017). AC6 overexpression prevents cardiac hypertrophy, fibrosis, and cardiomyopathy (Roth et al., 1999; Tang et al., 2013), while cardiac-directed expression of catalytically inactive AC6 restored the detrimental effects of sustained catecholamine infusion, through diminished myocardial cAMP production (Gao et al., 2017). In addition, in ischemic cardiomyopathy, cAMP production and systolic and diastolic LV function are enhanced after activation of AC6 expression (Lai et al., 2008). AC6KO mice exhibit reduced cAMP production and have significantly higher mortality compared to AC6WT, but remain susceptible to βAR stimulation-induced cardiomyopathy, compromised electrophysiological characteristics including diminished longitudinal conduction velocity, and impaired connexin 43 phosphorylation at Ser 368, which may be part of the mechanism of ventricular dysfunction (Tang et al., 2013). Cardiac-directed AC6 overexpression with Gαq expression triggered improved βAR-stimulated AC activity, cAMP generation, and cardiac function *in vivo* and *ex vivo,* with no sign of hypertrophy and fibrosis (Roth et al., 1999), as well as improved Ca^2+^ handling and LV contractility manifested by ejection fraction, pressure development rate, and slope of the LV end-systolic pressure–volume relationship in aging mice (Tang et al., 2011). In 23-month-old rats, AC6 expression improved SR Ca^2+^ storage, while AC6 expression in 7-month-old mice did not show any difference in LV function and Ca^2+^ uptake (Tang et al., 2011). While cardiac troponin I (cTnI) phosphorylation diminishes with cardiac age (Jiang et al., 1993), improved LV function by AC6 is associated with phosphorylation of cTnI at Ser 23/24, which regulates thin filament function and thus contractility (Lai et al., 2008; Tang et al., 2011). While AC6 expression increases PKA activity and SR Ca^2+^ uptake (Tang et al., 2011), deletion of AC6 results in diminished PKA activity, phospholamban (PLB) phosphorylation, and decreased SR Ca^+2^-ATPase activity 2a (SERCA2) affinity toward Ca^+2^ in failing hearts (Takahashi et al., 2006; Tang et al., 2008). Increased AC6 content increases the expression of activating transcription factor-3, which extinguishes PLB promoter activity, resulting in reduced PLB expression (Gao et al., 2004).

**FIGURE 8:**
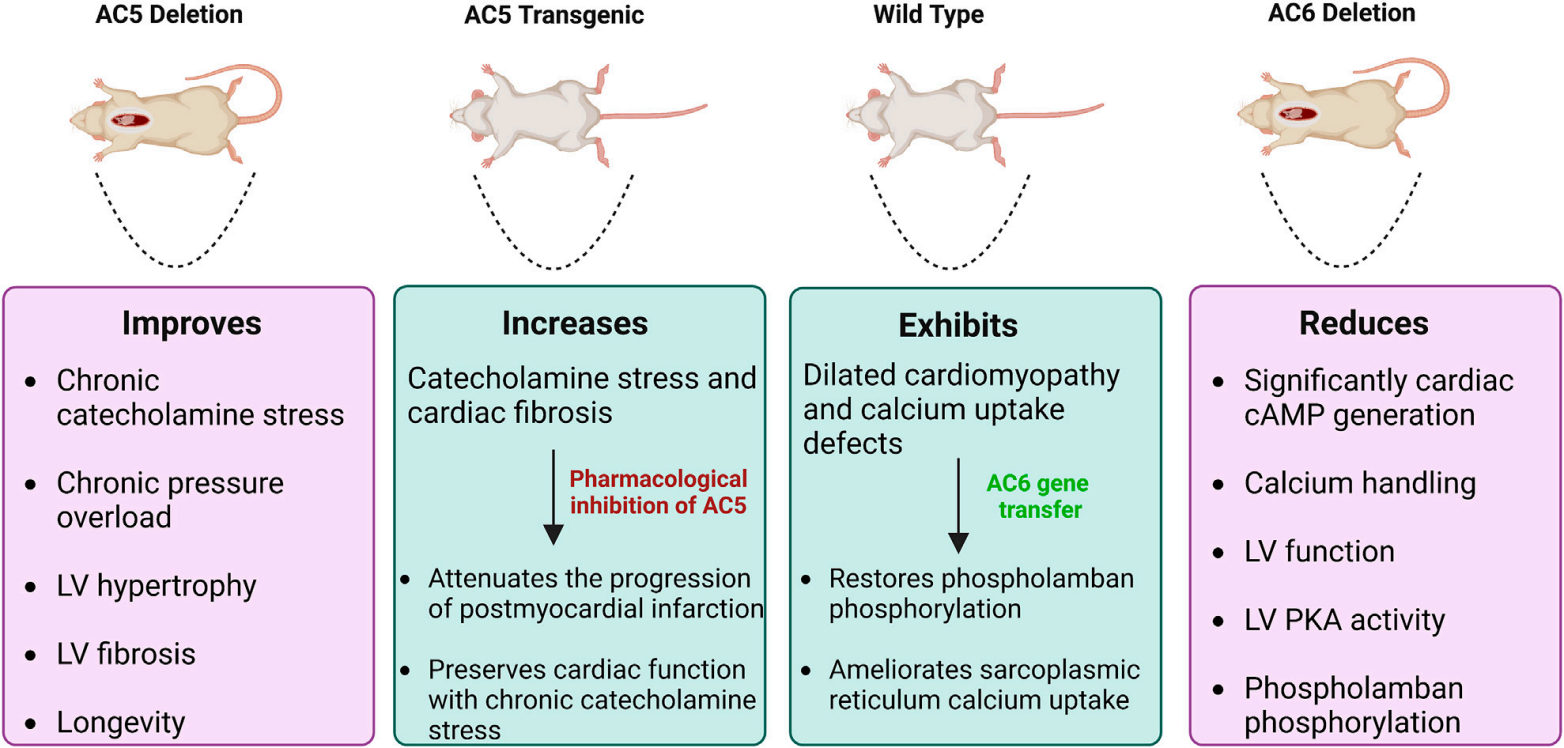
Effects of AC5 and AC6 overexpression or deletion on heart diseases *in vivo*. WT and transgenic mice subject to heart failure by catecholamine stress. Mice with deletion of AC5 demonstrate beneficial effects, whereas AC6 deletion is deleterious. AC5 transgenic mice have increased catecholamine stress and cardiac fibrosis. AC5 inhibitors rescue this condition, decreasing the progression of heart disease. Dilated cardiomyopathy in WT mice can be recovered by AC6 gene transfer.

Enhanced AC6 expression is also correlated with increased nuclear phospho-Akt promoting phosphorylation of Akt at Ser473 and Thr308. This process appears to be independent of PKA or βAR stimulation (Gao et al., 2008). Akt activity is reversely regulated by PH domain leucine-rich repeat protein phosphatase (PHLPP), responsible for dephosphorylation of Akt at Ser473. AC6 inhibits PHLPP activity in cardiomyocytes, bringing about high levels of Akt phosphorylation. This PHLPP suppression is, however, rescued rapidly by isoproterenol and FSK stimulation, leading to significant dephosphorylation of Akt at Ser473, but not Thr308. As PLB is an Akt target, active phospho-Akt increases PLB activity and thus improves sarcoplasmic Ca^2+^ cycling (Gao et al., 2009). Figure 8 gives an illustrated summary of the effects of AC5 and AC6 addition or deletion *in vivo*.

As AC6 shows remarkable physiological benefits for the heart, while its content and function are diminished in the failing heart (Ostrom et al., 2022), there has been increased interest in the efficacy and safety of adenoviral delivery of AC6 in patients with heart failure (Hammond et al., 2016) (AC6 Clinical Trials). In a mouse model, the AC6 C_1_–C_2_ construct was shown to effectively improve cardiac dysfunction caused by prolonged βAR stimulation (Roth et al., 2002). Although AC6 transgenic mice with cardiac-directed C_1_–C_2_ expression exhibited less cAMP production, they preserved normal cardiac function through ameliorated Ca^2+^ handling and tolerated sustained isoproterenol infusion and pressure overload without detrimental effects on LV contractility (Gao et al., 2016; Tan et al., 2019). Preclinical analysis of AC6 gene therapy recently transitioned into clinical trials. The phase 2 results for one-time AC6 gene transfer in 56 adult patients with symptomatic heart failure (ischemic or non-ischemic) and an ejection fraction of 40% resulted in safely ameliorated LV function above standard heart failure therapy (Hammond et al., 2016). The subsequent FLOURISH trial, a double-blinded placebo-controlled, multicenter phase 3 trial of 536 patients, aims to decrease heart failure hospitalization rates and improve ejection fraction, while minimizing adverse events after intracoronary injection of the human adenovirus 5 encoding human AC6 (Ad5. hAC6) gene in patients with heart failure and diminished LV ejection fraction; patient recruitment is ongoing (Penny et al., 2018).

Studies contextualizing the AC6 role in cardiovascular function are highlighted in Table 1.

## Roles of AC5 and AC6 in vasculature

AC activity in vascular cells regulates vascular reactivity, apoptosis, hypertrophy, and proliferation (Gros et al., 2006). The distribution of AC isoforms varies in the vasculature compared to the myocardium. Based on RT-PCR studies, ACs 3, 5, and 6 are expressed in the adult rat aorta (Ostrom et al., 2002), while in the perinatal period, the ductus arteriosus expresses more AC2 and 6 than does the aorta (Yokoyama et al., 2010). The pulmonary artery expresses AC6 primarily, followed by ACs 7, 9, and 3 (Sikarwar et al., 2018). In vascular myocytes, AC6 is the main AC isoform involved in βAR-mediated cAMP/PKA signaling and activation of the K_ATP_ current, important for harmonizing the membrane potential and regulating vascular tone (Nelson et al., 2011). Subjects who carry the genetic variation *ADCY6 A674S* have increased blood pressure, with a hyperdynamic cardiac profile that is compatible with the effect of elevated AC function (Hodges et al., 2010). Vasodilation is antagonized by Gαq-mediated signaling, in part by direct Ca^2+^ inhibition of AC5 and AC6 (von Hayn et al., 2010).

**FIGURE 9:**
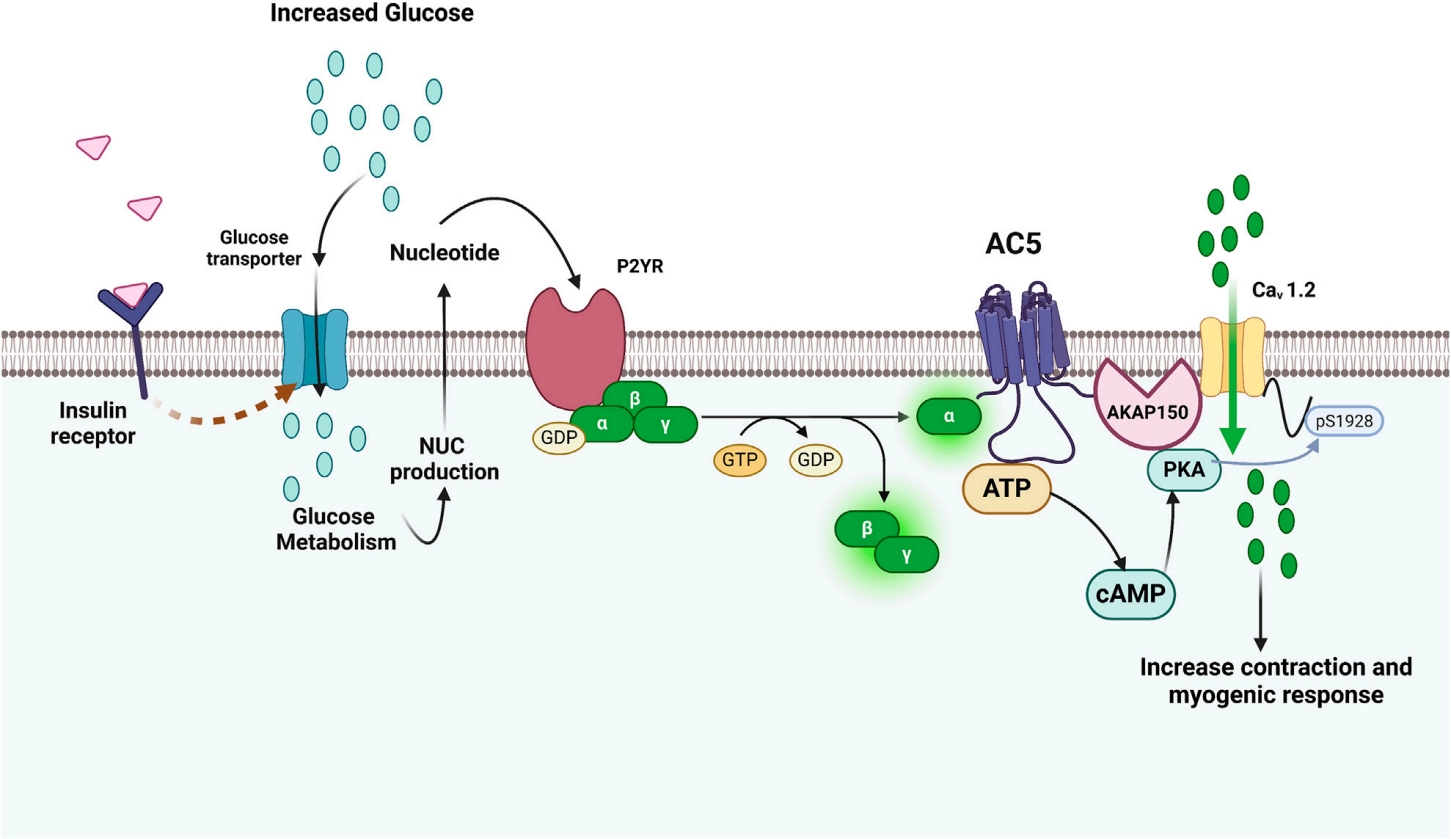
Involvement of AC5-mediated localized cAMP generation in diabetes and extracellular glucose activation of L-type Ca2+ channels and vasoconstriction. The illustration shows how glucose regulates L-type Ca^2+^ channel activity and vascular reactivity in an AC5-dependent manner. Increases in extracellular glucose can trigger P2YR linked to Gαs signaling through extracellular nucleotide signaling because of transport and metabolism. Because AC5 and Ca_V_1.2 are close in proximity, this cAMP microdomain may stimulate a pool of AKAP150-anchored PKA that is jointly linked to Ca_V_1.2, making it to become more phosphorylated at Ser1928 and increasing channel function. Adapted from (Syed et al., 2019); image designed using BioRender.

Changes in AC activity have been related to the development of diabetes, heart failure, and hypertension (Matsumoto et al., 2005; Hodges et al., 2010). Abnormal arterial myocyte contractility, in addition to compromised endothelium-dependent vasodilation, is an important contributing factor to vascular complications including altered myogenic tone, both in diabetic mice and in patients with diabetes (Montero et al., 2013; Sena et al., 2013; Tykocki et al., 2017; Syed et al., 2019). The contractile state of smooth muscle cells in the vessel wall determines the vascular tone, measured by a balance between the effects of vasoconstrictor and vasodilator signaling pathways, inclusive of adrenergic receptors and ACs (Shi et al., 2020). Physiological targets of ACs and downstream PKA include potassium channel phosphorylation, inducing hyperpolarization and vasodilation (Nelson et al., 2011). In resistance arteries and arterioles, the myogenic tone generated through the pulsatile stretch of the vascular wall modulates baseline smooth muscle contraction (Tykocki et al., 2017); in diabetic hyperglycemia, altered expression or function of potassium channels is linked to increased myogenic tone (Syed et al., 2019). Elevated glucose can trigger Gαs signaling (Lemaire et al., 2004) but may drive vasoconstriction through glucose-induced cAMP production via AC5, which results in the activation of an anchored PKA pool, and in turn, phosphorylates the L-type Ca^2+^ channel pore-forming Ca_V_1.2 subunit at Ser 1928 (Nystoriak et al., 2017; Prada et al., 2019; Syed et al., 2019). These interactions cause potentiation of L-type Ca^2+^ channel activity, enhanced [Ca^2+^]_i_, and vasoconstriction (Figure 9). Supporting this, interruption of AKAP5 function in arterial myocytes prevents cAMP generation in response to either increased glucose or selective purinergic P2Y11 agonist NF546; in AKAP5-null arterial myocytes or arteries, there is no clustering of P2Y11/P2Y11-like receptors, AC5, PKA, and Ca_V_1.2 into nanocomplexes at the plasma membrane; therefore, glucose- and NF546-induced potentiation of L-type Ca^2+^ channels and vasoconstriction does not occur (Prada et al., 2020). These data implicate AKAP5 and AC5 in the spatial confinement of cAMP signaling induced by elevated glucose via activation of P2Y11/P2Y11-like receptors in arterial myocytes (Prada et al., 2019; Prada et al., 2020).

ACs in endothelial cells play a role in vascular permeability. Prostacyclin-mediated signaling via AC6 (but not AC5) forms part of a feedback circuit that increases endothelial barrier function. Endothelial cells overexpressing AC6 have increased prostacyclin response, reducing the permeability of the endothelial barrier. In human umbilical vein endothelial cells, adenoviral-mediated gene transfer of AC6 increased prostacyclin receptor-stimulated cAMP synthesis and concurrently decreased thrombin-stimulated increases in endothelial cell barrier function (Bundey and Insel, 2006).

## Role of ACs in cardiac automaticity

The sinoatrial node (SAN) is a crescent-like shaped cluster of myocytes split by connective tissue, spread over a few millimeters (Kashou et al., 2017), located at the convergence of the superior vena cava opening and the crista terminalis in the upper wall of the right atrium. The SAN comprises coordinated actions of pacemaker cells capable of generating a cyclic electrical impulse (Kashou et al., 2017). Cardiac arrhythmia due to the abnormality of the SAN can affect annually up to 1 per 1000 adults >45 years of age (John and Kumar, 2016). The SAN is the main regulator of heart rate and is regulated by βAR signaling (Irisawa et al., 1993; Tsutsui et al., 2021). While the expression of a number of AC isoforms (types 1, 2, 3, 5, 6, 8, and 9) has been identified in rabbit SAN, AC1 and AC8, two of the Ca^2+^-activated AC isoforms, are predominantly distributed in atrial and SAN cells (Mattick et al., 2007; Younes et al., 2008; Robinson et al., 2021). In contrast, a recent study showed AC1 and AC6 but not AC8 expressed in SAN cells at the transcript and protein levels (Ren et al., 2022).

Ca^2+^ is a vital modulator of pacemaker potential via the Ca^2+^ clock (Maltsev and Lakatta, 2012), where the ryanodine receptor 2 (RyR2) facilitates the spontaneous release of Ca^2+^ from the SR, which in turn pushes Ca^2+^ to be released from the cytosol via the Na^+^–Ca^2+^ exchanger (Maltsev and Lakatta, 2012; Ren et al., 2022). Adrenergic control of the cardiac pacemaker current has been ascribed to AC1 (Mattick et al., 2007). AC1 mediates cAMP signaling in the SAN, in a functional microdomain with CAV3, hyperpolarization-activated cyclic nucleotide-gated 4 (HCN4), Ca_v_1.2, and RyR2. cAMP released by AC1 leads to elevation of intracellular Ca^2+^ via Ca^2+^ channels, which triggers a positive feedback to AC1 and negative feedback to AC5/6 (Ren et al., 2022). While cardiac-specific overexpression of AC8 transgenic mice in the SAN is reported to augment the heart rate and rhythm (Moen et al., 2019), others found no differences in automaticity, basal heart rate, or isoproterenol responses in AC8-null mice compared with wild-type (Ren et al., 2022). In guinea pig atrial myocytes, sarcoplasmic reticulum type 2 inositol trisphosphate (IP3) receptors colocalize with AC8, with AC1 localized proximally. Functional AC1 and AC8 are required for the positive chronotropic effect of phenylephrine on the SAN, and activity of both AC1 and AC8 plus PKA is required for the effect of IP3 on cellular Ca^2+^ transients (Capel et al., 2021).

Pacemaker current is generated by hyperpolarization-activated cyclic nucleotide-gated channels (HCN). To assess the role of Ca^2+^ homeostasis in autonomic regulation, AC1 and AC6 were expressed in cultures of spontaneously beating neonatal rat ventricle cells co-expressing HCN2. AC1, but not AC6 expression, increased intracellular cAMP and automaticity; AC1-mediated cAMP generation was resistant to β-adrenergic blockade, but the HCN2 response to an adrenergic agonist in the presence of AC1 (but not AC6) was sensitive to Ca^2+^ chelation, implying that the effect of Ca^2+^ homeostasis on the adrenergic regulation of the pacemaker rate could be accounted by the existence of a Ca^2+^ sensitive AC isoform (Kryukova et al., 2012; Robinson et al., 2021). However in a study of *in vivo* adenoviral gene transfer of AC6 in a porcine atrioventricular node block model, the AC6 injected group developed an escape rhythm of ∼100 beats/min originating at the LV injection site, while control animals had RV escape rhythms, suggesting that biological pacemaker activity could also be triggered by AC6 (Ruhparwar et al., 2010).

## AC5 and AC6 as potential drug targets for cardiovascular disease

Although βAR agonists and antagonists have therapeutic effectiveness for heart failure treatment, there are still patients who do not respond effectively; thus, heart failure is the most common cause of mortality worldwide (Capote et al., 2015). A weak cardiac response to catecholamine stimulation is a characteristic of the heart failure phenotype; hence, stimulation of the adrenergic pathway has been targeted to increase cardiac function. However, βARs can undergo downregulation after prolonged stimulation by agonists or antagonists, which leads to a reduction in their cell surface density (tachyphylaxis), modifications in subtype composition, or increase in PKA or G-protein-coupled receptor kinase (GRK) activity, resulting in uncoupling of βAR from G proteins (desensitization) (Mahmood et al., 2022). βAR desensitization also serves as a compensatory mechanism, which may disrupt cardiac function and promote arrhythmias (Ho et al., 2010).

In contrast to GPCRs, AC5 and AC6 are less likely subject to desensitization after sustained ligand exposure (Pierre et al., 2009). The AC5 and AC6 isoforms have been indirectly targeted by GPCR agonists or antagonists and PDE inhibitors due to their action as the central relay site that assembles and amplifies a wide variety of signals (Pavan et al., 2009). AC5 and AC6 have pivotal roles in cardiac disease. Despite the promising results from knockout and transgenic mice, suggesting that AC5 and AC6 can be potential drug targets, advancements to selectively and therapeutically target these isoforms have been hindered by their structural similarity. However, many positive steps in this direction are reviewed below.

### AC5 and AC6 activators for the treatment of heart failure

The most potent stimulus for increasing the cardiac output is initiated by sympathetic nervous system activation through the activation of βAR and Gαs, which in turn activates AC5 or AC6 to enhance cardiac contractility and rate. In the failing heart, production of basal and stimulated cAMP is diminished, but driving cardiac cAMP levels via adrenergic agents can result in higher long-term mortality (Bristow et al., 1982; Packer et al., 1991). FSK is a well-known activator of all transmembrane ACs, but its lack of selectivity impedes its clinical application; in addition to ACs, it also targets nuclear receptors, ion channels, glucose transporters, and P-glycoprotein multidrug resistance transporters (Dessauer et al., 2017). Clinically, in patients with heart failure, forskolin decreases ventricular filling pressures and vascular resistance while increasing cardiac output, ejection fraction, and stroke volume (Bristow et al., 1984). However, novel FSK-derived compounds have been synthesized that selectively target AC5 and AC6 isoforms (Pavan et al., 2009).

**FIGURE 10:**
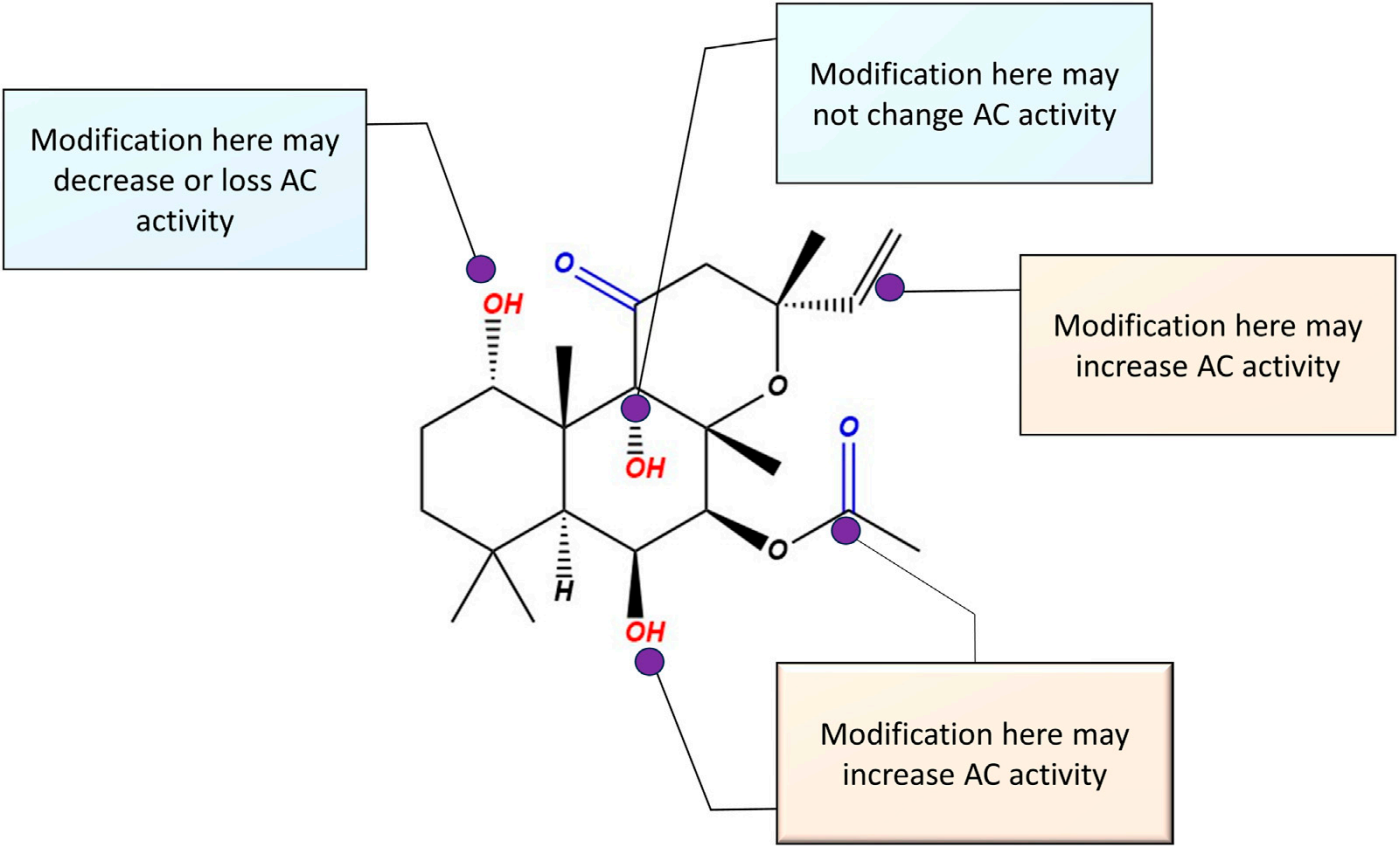
Possible modification sites on the parent compound forskolin molecule, for the design of selective AC5 or AC6 activators. Based on previous studies, any modification on C (1) reduces AC activity. Modifications on C (6) and (7) have the potential to enhance AC5 and AC6 activity.

To design selective AC5 and AC6 activators, maintaining hydrogen bonding of FSK C1-OH, C7-acetyl, and C9-OH has been deemed crucial (Onda et al., 2001). There has also been specific focus on modification of C6 and C7 positions, due to a large open area apparent from AC crystallographic studies (Tesmer et al., 1997), suggesting that modification on these FSK positions may increase AC activity with selectivity and/or specificity. Figure 10 illustrates the possible sites of modifications for designing novel AC5 or AC6 activators. It is important to note that many of the earlier studies which established FSK positions suitable for modification evaluated only a subset of the AC isoforms. Testing of candidate compounds on each AC isoform remains necessary to determine which site is particular to each isoform.

The development of AC5 activator 6-[3-(dimethylamino)propionyl]FSK (NKH477, FD5, or colforsin daropate hydrochloride) (Toya et al., 1998) grabbed the attention of scientists working on AC drug discovery. Colforsin is a potent water-soluble derivative of the C6 position of FSK, initially approved in Japan for the treatment of advanced congestive heart failure due to the elevation of cAMP in cardiac tissue, resulting in the enhancement of cardiac contractility. From the standpoint of AC isoform selectivity, colforsin activates AC5 > AC2∼AC3 (Toya et al., 1998). Using the isolated perfused canine heart, the chronotropic, inotropic, coronary vasodilatory effects, and AC activity of colforsin were compared to those in catecholamines isoproterenol, dopamine, and dobutamine. All the drugs demonstrated positive chronotropic, inotropic, and coronary vasodilator effects. Colforsin cardiovascular actions were in the following order: coronary vasodilation >> positive inotropy > positive chronotropy, while isoproterenol, dopamine, and dobutamine evinced positive inotropy >> coronary vasodilation > positive chronotropy. While inotropic effects are desirable, chronotropic agents have potential arrhythmogenic effects and may impair cardiac filling; however, at doses with comparable inotropic effects, colforsin demonstrated a higher positive chronotropic effect than catecholamines or PDE inhibitors, with ventricular tachycardia triggered equally by colforsin and isoproterenol. Nevertheless, the coronary vasodilator activity of colforsin was more potent. The strong coronary vasodilator function of colforsin may therefore be applicable for the treatment of heart disease where coronary blood flow is limited and βAR-dependent signaling is also downregulated (Yoneyama et al., 2002). In a model of canine respiratory acidosis causing cardiac dysfunction, colforsin increased cardiac rate and decreased systemic vascular resistance to the same degree as did dobutamine, without attenuation by acidosis (Itami et al., 2019). Colforsin has also demonstrated pulmonary artery vasodilation (Yokochi et al., 2010), which is mediated largely through AC6 rather than AC5 (Sikarwar et al., 2018), indicating that colforsin may not be AC5-selective (Jaggupilli et al., 2018). 6-[3-(Dimethylamino)propionyl]-14–15-dihydro-FSK (FD6) is another FSK derivative, structurally similar to colforsin, but with reduced 14–15 alkyne bond, that showed AC5 and AC6 selectivity (Yokoyama et al., 2010). The lack of complete AC5 *versus* AC6 selectivity of colforsin and similar compounds does complicate our understanding of isoform-specific cardiac effects of AC activation, in the absence of more preclinical data. Protective or detrimental effects of individual AC manipulations on cardiac function can perhaps be best deciphered from gene overexpression or deletion studies; precise pharmacological interrogation of AC5 or AC6 activation would require further development of more selective tools.

**FIGURE 11:**
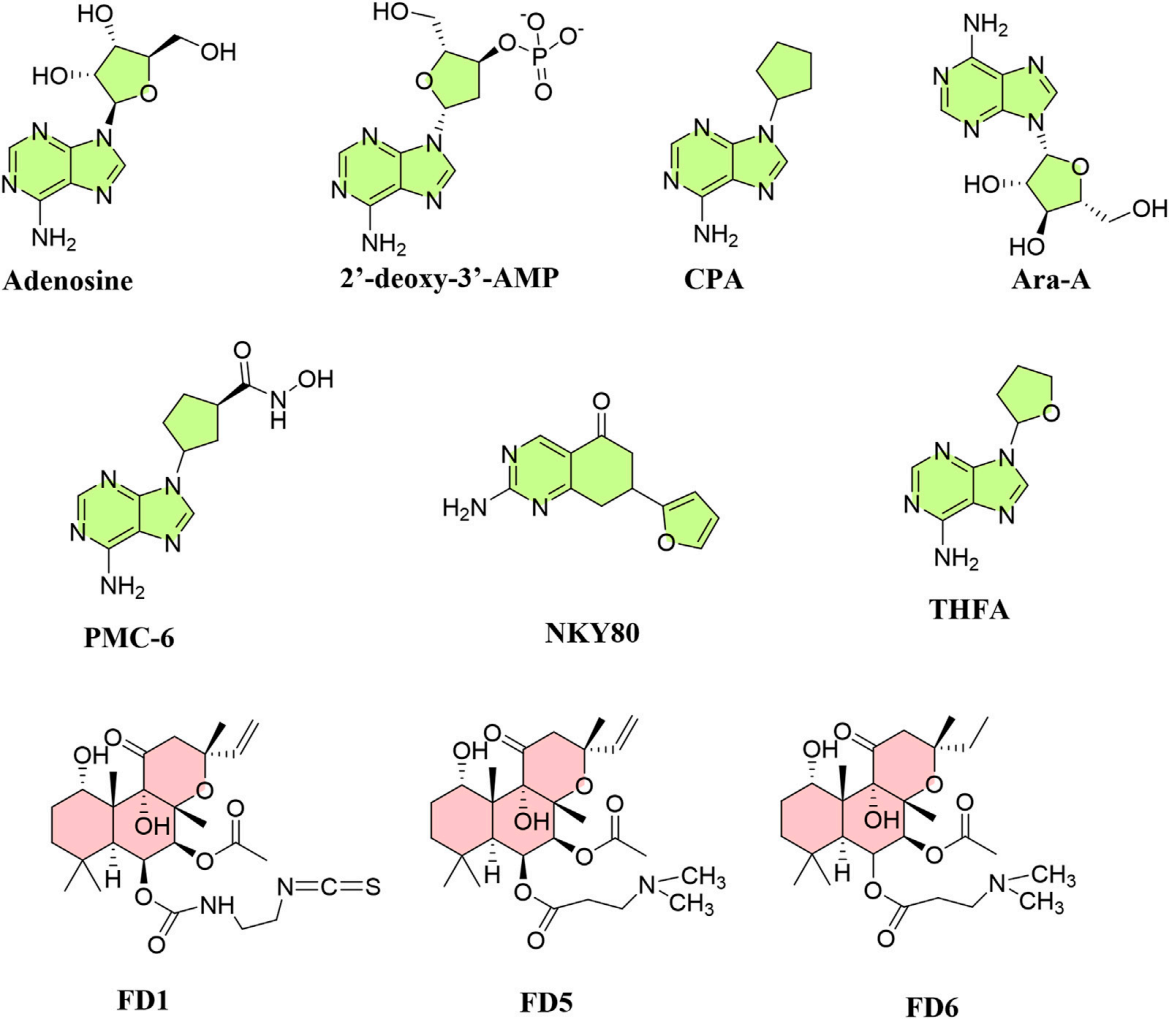
Structures of known inhibitors and activators targeting AC5 and AC6 allosteric or catalytic sites. Inhibitors (green) are mainly P-site inhibitors, and activators (red) are forskolin derivatives.

A list of published inhibitors and FSK derivatives having AC5 and/or AC6 selectivity is depicted in Figure 11.

### AC5 and AC6 inhibitors for the treatment of heart failure

As AC5 deletion appears to be protective in heart failure, there has been much research into isoform-selective AC5 inhibitors, which may be advantageous over β-blockers (Pierre et al., 2009). AC inhibitors are classified into two categories: competitive inhibitors [(M)ANT- and TNP-nucleotides] and non-competitive P-site inhibitors. Among AC5 and AC6 inhibitors discovered, 2′-d-3′-MANT-GDP has inhibitory effects on AC6 (∼8-fold) compared to AC5 (Gille et al., 2004). Among P-site inhibitors with metal chelating characteristics (PMC), PMC-6 has shown selectivity for AC5, protecting cardiac myocytes from βAR-induced apoptosis without degrading cAMP synthesis or contractility (Iwatsubo et al., 2004). P-site inhibitors that are adenosine analogs interact with ACs when PP_i_ is present and act by obstructing the interaction of other substrates with P-sites (Dessauer and Gilman, 1997). Unlike FSK, P-site inhibitors attach to the catalytic site for substrate ATP (Tesmer et al., 2000), generating a dead-end complex with PPi (Bushfield et al., 1990). Ribose-substituted P-ligands such as 9-(cyclopentyl)-adenine (CPA) and 9-(tetrahydrofuryl)-adenine (THFA) have IC_50_ values in the micromolar range, selectively inhibiting AC5 more than AC3 and AC2; while 2′-deoxy-3′-AMP and 3′-AMP inhibit AC3 and AC5 more than AC2 (Onda et al., 2001; Iwatsubo et al., 2003). 2-Amino-7-(2-furanyl)-7,8-dihydro-5(6H)-quinazolinone (NKY80), derived from 9-(tetrahydro-2-furanyl)-9H-purin-6-amine (SQ22,536), like 9-(tetrahydro-2-furyl)adenine (THFA), inhibits AC5 despite not possessing an adenine ring. NKY80, though less potent, exhibits a similar AC5 selectivity to THFA in the inhibition of AC5 catalytic activity, having a selectivity ratio of 210 between AC5 and AC2 with an IC50 of 8.3 μM for AC5, 132 μM for AC3, and 1.7 mM for AC2 when Gαs–GTPγS–forskolin is present (Onda et al., 2001).

The antiviral drug adenine 9-β-D-arabinofuranoside (Ara-A), known as vidarabine, has been found to selectively inhibit AC5; Ara-A markedly diminished AC activity in AC5 transgenic mice, but not in AC5KO, and had a minor effect in either WT or AC6 transgenic mice (Iwatsubo et al., 2012). However, another study indicated that Ara-A is also a potent AC6 inhibitor (pIC_50_: 5.67 and 5.34, for AC5 and AC6, respectively); indeed, SQ22,536, NKY80, and Ara-A inhibit both AC5 and AC6 without distinguishing them (Brand et al., 2013). Inhibition of AC5 by Ara-A is thought to be mediated through the MEK/ERK pathway. The Ca^2+^-binding protein annexin A4 (ANXA4) selectively inhibits AC5. Both ANXA4 and a peptide encompassing the ANXA4 N-terminal sequence (A4N_1-22_) reduced cAMP generation in AC5; ANXA4 co-immunoprecipitates with AC5 but not AC6, binding to its N-terminal domain. The peptide A4N_1-22_ diminishes recruitment of the L-type Ca^2+^ current (I_CaL_) and prevents action potential prolongation after catecholamine challenge, an effect similar to the loss of the β1AR signal in AC5KO models (Heinick et al., 2020). Another novel AC5 inhibitor, C90, inhibits cAMP production to FSK by 42% in WT but not in AC5KO, suggesting its selective AC5 inhibitory effect; it is five times less potent in inhibiting AC2 and AC6. C90 also produced the interesting effect of decreasing myocardial infarct size even when administered after coronary reperfusion (Zhang et al., 2018).

## Conclusion

Although there have been a number of AC5 and AC6 activators/inhibitors tested *in vitro*, more *in vivo* studies are required for evaluation of the cardiac AC isoforms as drug targets and more precision of selectivity testing, given the similar structures and expressions patterns of cardiac ACs. Advances in detailed structural information (Qi et al., 2019) as well as computational homology modeling and mutational analysis of ligand-binding regions (Bhatia et al., 2023) will provide further direction to drug discovery for selective targeting of cardiovascular ACs, as will the selection of appropriate pharmacokinetic features and targeted drug delivery systems to decrease off-target adverse effects.

The prevalence of cardiovascular disease is on the rise, despite the rapid advancements in drug discovery for therapeutics addressing the alteration in the adrenergic signaling profile in the failing heart. Increased activity of AC6 has a beneficial effect on cardiac contractility, cell survival, and Ca^2+^ handling, while the activation of AC5 is deleterious. Differentiating the regulation of AC5 and AC6 and their signaling pathways opens up potential avenues for selective manipulation of physiologically important cAMP pools. The development of AC5/6 selective activators or inhibitors is still in its infancy, generating novel stimulators and inhibitors or taking advantage of drug repositioning; AC6 gene transfer also holds promise for heart failure treatment. Further research in these directions is clearly warranted.
